# Bioinformatics Analysis of Alternative Polyadenylation in Green Alga *Chlamydomonas reinhardtii* Using Transcriptome Sequences from Three Different Sequencing Platforms

**DOI:** 10.1534/g3.114.010249

**Published:** 2014-03-13

**Authors:** Zhixin Zhao, Xiaohui Wu, Praveen Kumar Raj Kumar, Min Dong, Guoli Ji, Qingshun Quinn Li, Chun Liang

**Affiliations:** * Department of Biology, Miami University, Oxford, Ohio 45056; † Department of Automation, Xiamen University, Xiamen, 361005, China; ‡ Key Laboratory of the Ministry of Education for Coastal and Wetland Ecosystems, and College of the Environment and Ecology, Xiamen University, Xiamen, Fujian, China 361102; § Rice Research Institute, Fujian Academy of Agricultural Sciences, Fuzhou, Fujian, China 350003

**Keywords:** poly(A) signals, alternative polyadenylation, alternative splicing, intron retention, sequencing platforms, *Chlamydomonas reinhardtii*

## Abstract

Messenger RNA 3′-end formation is an essential posttranscriptional processing step for most eukaryotic genes. Different from plants and animals where AAUAAA and its variants routinely are found as the main poly(A) signal, *Chlamydomonas reinhardtii* uses UGUAA as the major poly(A) signal. The advance of sequencing technology provides an enormous amount of sequencing data for us to explore the variations of poly(A) signals, alternative polyadenylation (APA), and its relationship with splicing in this algal species. Through genome-wide analysis of poly(A) sites in *C. reinhardtii*, we identified a large number of poly(A) sites: 21,041 from Sanger expressed sequence tags, 88,184 from 454, and 195,266 from Illumina sequence reads. In comparison with previous collections, more new poly(A) sites are found in coding sequences and intron and intergenic regions by deep-sequencing. Interestingly, G-rich signals are particularly abundant in intron and intergenic regions. The prevalence of different poly(A) signals between coding sequences and a 3′-untranslated region implies potentially different polyadenylation mechanisms. Our data suggest that the APA occurs in about 68% of *C. reinhardtii* genes. Using Gene Ontolgy analysis, we found most of the APA genes are involved in RNA regulation and metabolic process, protein synthesis, hydrolase, and ligase activities. Moreover, intronic poly(A) sites are more abundant in constitutively spliced introns than retained introns, suggesting an interplay between polyadenylation and splicing. Our results support that APA, as in higher eukaryotes, may play significant roles in increasing transcriptome diversity and gene expression regulation in this algal species. Our datasets also provide useful information for accurate annotation of transcript ends in *C. reinhardtii*.

Messenger RNA (mRNA) 3′-end formation, including cleavage and polyadenylation, is a crucial step in posttranscriptional processing of eukaryotic mRNAs. Polyadenylation plays essential roles for eukaryotes, including protection of mature mRNAs from unregulated degradation and recognition by mRNA cytoplasm export machinery and by translational apparatus. Moreover, the formation of 3′-end is indispensable for transcription termination (Rothnie 1996; Zhao *et al.* 1999; Hunt *et al.* 2012).

As a sequenced model organism, unicellular green alga *Chlamydomonas reinhardtii* is widely used in cell, molecular, and evolutionary biology research and is also used in biopharmaceutical and hydrogen production. *C. reinhardtii* diverged from the streptophytes more than a billion years ago and shared lots of plant-animal common ancestor genes with animals (Merchant *et al.* 2007). Therefore, the poly(A) study in *C. reinhardtii* will enhance our understanding of polyadenylation in eukaryotes. Apparently different from most higher eukaryotes, in which AAUAAA is believed to be the canonical poly(A) signal (Graber *et al.* 1999; Tian *et al.* 2005; Mangone *et al.* 2010; Wu *et al.* 2011), it was reported that UGUAA, a poly(A) signal found in the near upstream element region (NUE), plays vital functions in alpha 2- and beta 2-tubulin−encoding genes in green algae *Volvoxcarteri carteri* and *C. reinhardtii* (Mages *et al.* 1995). A bioinformatics analysis using 10,508 cDNA/expressed sequence tag (EST) data in *C. reinhardtii* revealed that ~50% of the sequences contain the UGUAA signal within 50 nt upstream from the cleavage sites (CS) or poly(A) site (Wodniok *et al.* 2007). Shen *et al.* (2008a) also reported that UGUAA is the most dominant poly(A) signal (~52% of 16,952 EST sequences) in the NUE region in *C. reinhardtii*.

In eukaryotes, many genes have more than one poly(A) site, and such distinctive sites would generate multiple mRNA isoforms with different sequence lengths and 3′-ends from the same genes. This phenomenon is called alternative polyadenylation (APA), which has been proved to play a crucial role in gene expression regulation. Examples of such regulation include cancer cell division control, neuronal cell development, immune responses in animals (Elkon *et al.* 2013), amino acid metabolism, self-incompatibility, RNA processing, flower time control (Xing and Li 2011), fungal disease resistance, and epigenome modifications in plants (Ma *et al.* 2014). In yeast *Saccharomyces cerevisiae*, APA also has been found to play a role in gene regulation through antisense transcripts (Ozsolak *et al.* 2010). However, information about the extent of APA is very limited in algae, except some work in *C. reinhardtii* (Shen *et al.* 2008a). This hampers the study of the potential roles of APA in algal gene expression.

Based on Sanger EST/cDNA data, the extent of APA in *C. reinhardtii* was shown to be up to 33% (Shen *et al.* 2008a). With significant increase of available transcriptomics data, one would expect that additional APA may be found. Indeed, the occurrence of APA is influenced by sequencing depth (Wu *et al.* 2011; Shen *et al.* 2011). For example, the extent of APA in rice is ~47% and 82% supported by using digital gene expression based protocols of the massively parallel signature sequencing and the Illumina sequencing-by-synthesis, respectively (Shen *et al.* 2011). Interestingly, alternative mRNA processing, particular APA, would lead to transcript isoform abundance variation and can be used as powerful molecular biomarkers in cancer research and diagnosis (Singh *et al.* 2009).

Not only APA can produce multiple isoforms from the same genes, but alternative splicing also can generate different transcripts from the same genes. Research shows that introns with poly(A) sites in human have weaker 5′ splice site and larger intron size comparing with the introns without a poly(A) site (Tian *et al.* 2007). A similar relationship between polyadenylation and splicing in introns is also detected in Arabidopsis (Wu *et al.* 2011). Moreover, intronic poly(A) signals are shown to reciprocally regulate splicing and polyadenylation and control sFlt1 (shorter Fms-like tyrosine kinase-1) gene expression (Thomas *et al.* 2007, 2010).

The chain-termination DNA sequencing method, also known as Sanger sequencing developed in 1975, was the dominant sequencing technology (Sanger and Coulson 1975; Sanger *et al.* 1977). Following the need for deciphering the human genome, capillary electrophoresis technology was developed and integrated with the chain-terminating fluorescence dye method in the late 1980s. Now ultra-high-throughput sequencing technologies, often referred to as next-generation sequencing (NGS), are rapidly replacing Sanger sequencing because they are revolutionizing the sequencing depths and coverage with much reduced cost (Schuster 2008; Mardis 2008). The first NGS technology, Roche/454 FLX Pyrosequencing using sequencing-by-synthesis technology, was developed by 454 Life Sciences and Roche Applied Science in 2005 (Margulies *et al.* 2005). Afterward, RNA-Seq (Wang *et al.* 2009) and direct RNA sequencing (DRS) (Ozsolak *et al.* 2009) technologies provided deeper transcriptomics data. The massive data accumulated over the last few years provide us with a great opportunity to examine the issues of polyadenylation and splicing in many eukaryotic species.

Although the poly(A) signal UGUAA has been found computationally and verified experimentally in *C. reinhardtii*, its variations in different genic regions (*i.e.*, 5′-untranslated region [UTR], 3′-UTR, CDS, and intron) and intergenic regions have not been systematically studied and compared. In this study, we compared the poly(A) sites obtained by different sequencing technologies and found that deep-sequencing (454 and Illumina sequencing) revealed much more previously unannotated poly(A) sites. In particular, a greater proportion of these new poly(A) sites are located in CDS, intron and intergenic regions. With the greater depth and coverage of the poly(A) site data, we now begin to realize the potential of APA in algal gene expression regulation. Moreover, our study suggested that polyadenylation is more likely to occur in constitutively spliced introns than retained introns in *C. reinhardtii*. Our datasets not only can be used for annotating gene/transcript ends accurately, but also provide useful information for further investigation of the regulatory role of APA in this model organism.

## Materials and Methods

### Data sources and polyadenylation site definition

Sanger-based EST sequences were collected from both JGI (US Department of Energy Joint Genome Institute) and The National Center for Biotechnology Information (NCBI) GenBank (http://www.ncbi.nlm.nih.gov/genbank/). Our collaborator (Dr. Olivier Vallon from Institut de Biologie Physico-Chimmique, Paris, France) provided us with 454 data in *C. reinhardtii*. In addition, more publically available 454 data and the most of Illumina RNA-Seq data were collected and downloaded from DNAnexus (http://sra.dnanexus.com/), a robust web portal that provides biologist-friendly search and browsing interfaces for NCBI Sequence Read Archive. For EST data, when available, raw EST trace files were used to identify poly(A)/(T) tails by identification of cDNA termini (Liang *et al.* 2008). Detailed information about data sources, poly(A) sites obtained, genome sequences, and gene annotation is listed in Table 1.

**Table 1:** The data sources and numbers of poly(A) sites from three sequencing platforms

Data Types	Source	No. of Reads With Poly(A) Tails	No. of Unique Poly(A) Sites	No. of Poly(A) Site Clusters
Genome sequences	Phytozome v4.3			
Gene annotation	Phytozome v4.3			
Expressed sequence tag data	National Center for Biotechnology Information, Joint Genome Institute, Liang *et al.* 2008	338,234	21,041	11,035
454 data	Collaborator, DNAnexus	824,565	88,184	30,086
Illumina data	DNAnexus	22,372,354	195,266	88,304
Total		23,535,153	256,771	97,479

For ESTs, the minimum length of valid poly(A) tails was set to be 10 nt. The maximum adenines that were integral part of poly(A) tails but also mapped to the genome were set to be 4 nt. The maximum nontemplated nucleotide addition (Jin and Bian 2004) between the poly(A) tails and poly(A) sites [*e.g.*, the nucleotides between the mapped end of the cDNA and the start of its poly(A) tail] was allowed to be 3 nt. All ESTs with identified poly(A) sites were mapped to the reference genome sequences with GMAP (Wu and Watanabe 2005). The EST-to-genome mapping results were then analyzed and filtered for valid genomic hits by the use of a similar protocol as described previously (Liang *et al.* 2008). For 454 and Illumina data, the poly(A) sites were identified using our in-house tool SCOPE++ (http://code.google.com/p/scopeplusplus/), with at least 15 nt in length and 95% identity in purity, and then the sequence reads with a valid poly(A) tail were mapped to the genome by the use of Helicos Heliosphere (Ozsolak *et al.* 2010). Because poly(A) tails detected in ESTs or RNA-Seq reads were posttranscriptional, they should not be mapped to the genome except in the case in which internal priming was likely to occur. The internal priming was defined as that there were at least 6−7 consecutive adenines (As) from 10 nt-window in −10 to +10 region around poly(A) sites in genomic sequences (Tian *et al.* 2005). Therefore, individual genomic poly(A) sites were finally determined through the sequence-to-genome mapping results. The poly(A) sites that might be due to internal priming were filtered out. All individual poly(A) sites were organized to generate a list of nonredundant, unique poly(A) sites with distinctive genomic coordinates.

It is well-known that polyadenylation complex appears to be “sloppy” when cleaving pre-mRNA before polyadenylation and therefore generates microheterogeneity in poly(A) sites (Tian *et al.* 2005; Shen *et al.* 2008a; Liang *et al.* 2008). To circumvent this, iterative clustering of adjacent unique poly(A) sites was performed in each chromosome. We developed an algorithm based on density theory and restraint theory in data mining to cluster poly(A) sites. In our algorithm, we used the advanced Ward’s minimum variance method (Szekely and Rizzo 2005), a popular hierarchical clustering method. To obtain high-quality poly(A) site clusters (PACs), all individual unique poly(A) sites with the support of at least three ESTs or RNA-Seq reads were used for clustering. These PACs were used to extract 100 nt upstream and 50 nt downstream sequences from the reference genome for further analyses [each poly(A) site is defined as −1 position in genomic coordinates (Shen *et al.* 2008a)]. The final PAC sites and relevant sequences extracted from the genome were saved in File S1.

### Poly(A) signal variation in different parts of the genome

In this study, the gene annotation (v4.3) from Phytozome (http://www.phytozome.net) was used to determine the regions of 3′-UTR, 5′-UTR, CDS, intron, and intergenic regions in *C. reinhardtii* genome. Furthermore, 3′-UTR regions were expanded to overcome potential incomplete annotation (Wu *et al.* 2011), so we can investigate the distribution of poly(A) sites in the immediate downstream of 3′-UTR region (namely the intergenic region) more accurately.

A new program, SignalSleuth2 (File S2), an improved version of SignalSleuth (Loke *et al.* 2005), was developed to perform exhaustive search of highly frequent signals within a defined region for the given sequence data. This program offers new functionalities including multiple scanning modes and Position-Specific Scoring Matrix. It also provides a signal distance optional parameter (-gap) if there is more than one signal in the sequence, called distribution scanning mode. Such mode can provide signal distribution in the given region, avoid missing counts for a specific motif that appears multiple times in that region, and prevent over-counting of overlapping motifs. For example, ATATATAT would represent once for ATATAT and TATATA motif search when the gap was set to be two. If we do not consider the distance (namely gap = 0), we called it an overlapping scanning mode. In addition, if there was more than one signal in a sequence, the program would choose the signal closest to the poly(A) site, this was called frequency scanning mode. For each mode, the program also provided Position-Specific Scoring Matrix results as one type of outputs. To check the statistic significance, we used Z-score to inspect the significance of the signals from Regulatory Sequence Analysis Tools (RSAT) based on Markov chain models (van Helden *et al.* 1998).

### APA analysis

Within a PAC, the unique poly(A) site that had the most sequence read support was treated as the representative site of this PAC. Therefore, PACs represented polyadenylation sites without microheterogeneity. Thus, APA genes were defined as those having at least two PACs, and non-APA genes should have only one PAC [which was also called constitutive poly(A) site]. For APA genes with multiple PACs, the PAC with 75% or more supporting reads was categorized as a strong poly(A) site, and other sites in the same gene were called weak poly(A) sites. If no strong site was detected in a APA gene, then all of the poly(A) sites were classified as median poly(A) sites (Hu *et al.* 2005). To investigate Gene Ontology (GO) functions for high-quality APA genes (*i.e.*, genes with at least five PACs), the GO annotation (v4.0) file of *C. reinhardtii* was downloaded from JGI. Then GOEAST (Gene Ontology Enrichment Analysis Software Toolkit) (Zheng and Wang 2008) was used to determine the enriched GO terms and their significance (p-value).

### The splicing events in *C. reinhardtii*

A total of more than 7 million *C. reinhardtii* cDNA sequences of both Sanger and NGS technologies were used to collect splicing events (P. K. Raj Kumar, O. Vallon and C. Liang). An automated version of PASA (Campbell *et al.* 2006) integrated with GMAP (Wu and Watanabe 2005) was used to deduce alternatively spliced isoforms. Seqclean (http://compbio.dfci.harvard.edu/tgi/software/) was used to trim off adapters, links, and poly(A)/(T) tails from raw sequence reads. Then, clean cDNAs reads were mapped to AUGUSTUS u10.2 gene annotation for *C. reinhardtii* with predicted protein mRNAs (Stanke *et al.* 2008) to define the mapped cDNAs as protein coding transcript fragments and the unmapped as non-coding transcript fragments. The results of protein coding and non-coding transcript fragments were separately used in PASA to deduce alternative splicing isoforms. All of the constitutively spliced introns (*i.e.*, introns that were spliced in all the isoforms) and retained introns (*i.e.*, introns that were retained in some isoforms but spliced in others) were extracted from the PASA database using Perl scripts, and the genes containing introns were then categorized into four different groups based on coding/noncoding and constitutive/retained introns (see Table 2).

**Table 2:** Number of PACs in different gene types

	Constitutive Introns		Retrained Introns	
	Protein-Coding Genes	Noncoding Genes	Protein-Coding Genes	Noncoding Genes
Intron number	134,708	2,550	5,151	116
No. of introns that contain PACs	12,031	153	120	5
% of introns that contain PACs	8.93%	6.00%	2.33%	4.31%

The percentage is calculated by (no. of introns that contains PACs)/(intron number). PACs, poly(A) site clusters.

## Results

### Poly(A) site collection and distribution in the *C. reinhardtii* genome

To study the extent of APA in the *C. reinhardtii* genome, we systematically acquired raw transcriptome sequence data from various sources (detailed in the section *Materials and Methods*). From 23,535,153 ESTs and RNA-Seq reads with poly(A) tails, a total of 256,771 unique poly(A) sites and 97,479 PACs were obtained after removing redundancy and potential internal priming candidates (Table 1).

**Figure 1:**
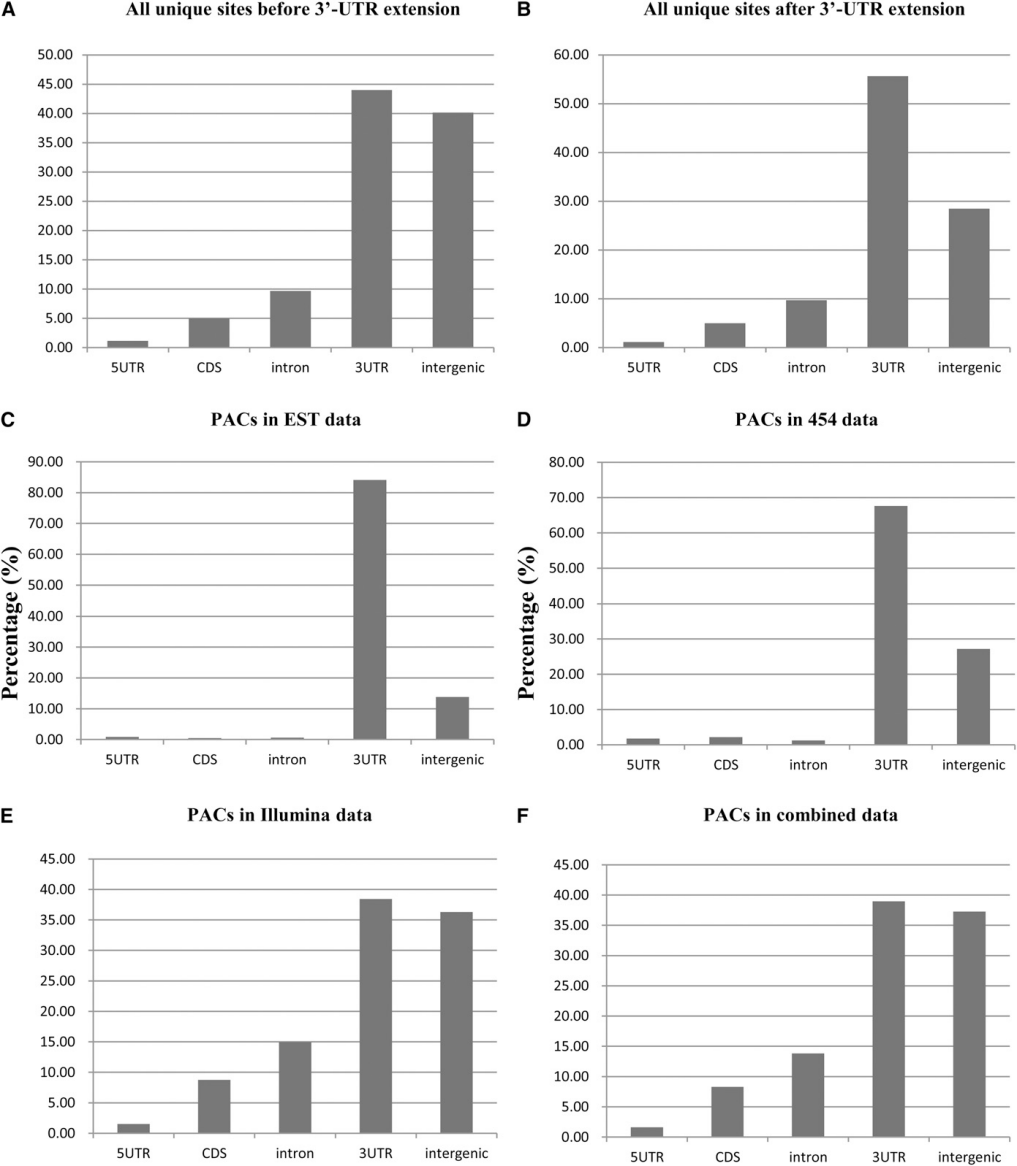
The distribution of poly(A) sites in the genic and intergenic regions in different datasets. (A) All unique poly(A) sites from Sanger expressed sequence tags (ESTs), 454, and Illumna before 50-nt extension. (B) All unique poly(A) sites from ESTs, 454, and Illumna after 50-nt extension. (C) Poly(A) site clusters (PACs) after 50-nt extension from EST data. (D) PACs after 50-nt extension from 454 data. (E) PACs after 50-nt extension from Illumina data. (F) PACs after 50-nt extension from all three combined data. UTR, untranslated region; CDS, coding sequences.

On the basis of *C. reinhardtii* genome annotation v4.3, we found that ~44% and 40% of unique poly(A) sites distributed in 3′-UTR and intergenic regions, respectively. The unique poly(A) sites in 5′-UTR, CDS and intron regions were approximately 1%~10% (Figure 1A). To circumvent potential genome annotation errors in 3′-UTR, the distribution of the intergenic poly(A) sites within 1000 nt to the 3′-UTR end of all genes was investigated (see Supporting information, Figure S1, File S1, File S2). It was found that there were many sites close to the annotated 3′-UTR ends. For example, there were 54% of the poly(A) sites located within 50 nt of the annotated 3′-UTR ends (see Figure S1). So, empirically 50 nt was used to expand 3′-UTR length to avoid annotation errors. This means that any poly(A) site located within 50 nt from the end of the annotated 3′-UTR will be treated as a poly(A) site in 3'-UTR of that gene.

### Single-nucleotide profiles and poly(A) signal variation

Single-nucleotide profiles around cleavage or poly(A) sites have been demonstrated to be a signature of poly(A) signals (Loke *et al.* 2005; Shen *et al.* 2008a,b). In general, there are three different groups of poly(A) signals in plants: the poly(A) site and its surrounding sequences called the cleavage element, the sequences from the NUE region of the poly(A) site, and the far upstream element (FUE) region from the poly(A) site (Loke *et al.* 2005). To analyze the poly(A) signals, 100 nt upstream and 50 nt downstream sequences for each PAC were extracted from the *C. reinhardtii* genomic sequences. We first examined the single-nucleotide profiles and mainly focused on the poly(A) signals in NUE and then FUE, because NUE is the most conserved region where the canonical poly(A) signal and its variants exist, and FUE also contained a group of nucleotide biased signals (Loke *et al.* 2005; Shen *et al.* 2008a,b; Xing and Li 2011).

**Figure 2:**
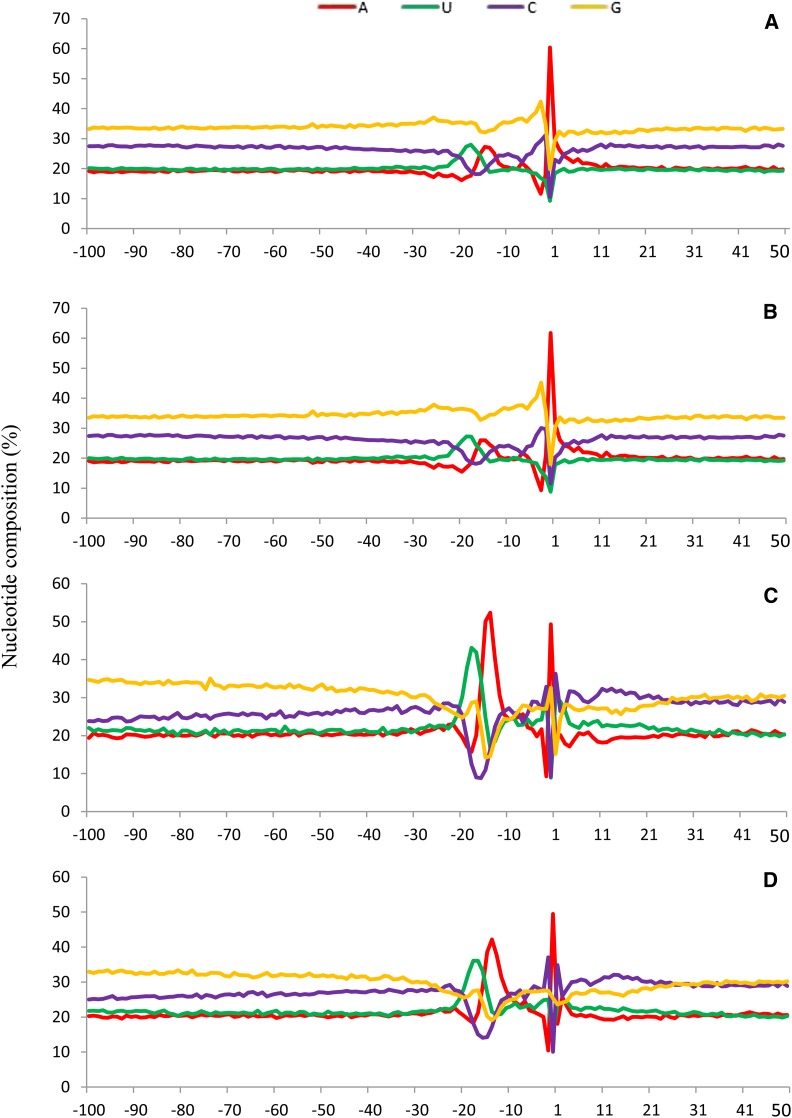
The single-nucleotide profiles from different datasets. (A) All combined poly(A) site clusters (including ESTs, 454 and Illumina). (B) Illumina data. (C) Expressed sequence tag data EST data. (D) 454 data.

The single nucleotide profile from all PAC data (Figure 2A) showed that the NUE region (around −28 to −5) had a high A-peak (60.39%) at the poly(A) site (−1 position), with a high G content (33.72% in average) in the whole investigated region (−100 to +50). Then each dataset from different sequencing platforms was specifically investigated, and it was shown that the difference is caused by Illumina data, because only the profile from Illumina has a high G content (34.24% in average) in the whole region (Figure 2B). The single nucleotide profiles from ESTs and 454 (Figure 2, C and D) were much similar to each other with normal G content (30.09% and 29.67% in average) in the whole regions, especially in the NUE regions (25.26% and 25.79% in average). Such a feature is much like what was described previously by Shen *et al.* (2008a). Regarding the NUE regions from the four profiles shown in Figure 2, it was clear that there were high U- and A-peaks and C- and G-troughs in *C. reinhardtii*.

Similar results were also obtained from the pentamer signals in the NUE regions among different datasets (Figure S2). The signal profiles from combined poly(A) site data show that GGGGG has the highest frequency (28.66%), but it cannot be significantly differentiated from the background noises due to a low Z-score (its Z-score was too low to be on the list generated by RSAT; Figure S2A). Pentamer UGUAA was found to be a significant (Z-score = 32.88 by using a third-order Markov model) and highly frequent (13.39%) signal in the NUE region, and G-rich pentamers GGUGG and GUGGG were also detected with significantly high frequencies and Z-scores (see Table S1 for details). Similar to all combined data, UGUAA and G-rich pentamers (*e.g.*, GGUGG and GUGGG) also were detected with high frequencies and Z-scores in the Illumina dataset (Figure S2B and Table S1). However, the pentamers from ESTs and 454 were similar to the results of Shen *et al.* (2008a), considering the UGUAA (>30%) and UGUAA-related motifs were dominant pentamers whereas G-rich pentamers are not found in those data (Figure S2, C and D, and Table S1). Such results indicated that Illumina data may be biased with overexpressed G nucleotide in sequencing or reveal a new polyadenylation feature in *C. reinhardtii*.

Among the FUE regions (around −100 to −28), G and C nucleotides had greater content than A and U nucleotides in the single nucleotide profiles (Figure 2). Consistently, the top detected pentamers were also GC-rich signals, especially in all combined and Illumina data (Figure S3, A and B, and Table S2). Although pentamer GUGUG always had the greatest frequencies (>21%) in all the datasets, yet it was not significant considering the Z-score (“None” means Z-score was too low to be on the list generated by RSAT). In the four datasets, the most frequent pentamers with significance were G-rich signals (*e.g.*, GGUGG, GUGCG, and GCGUG), especially in all combined and Illumina data (Figure S3 and Table S2); EST and 454 data had similar profiles and signals and no any single dominant signal (*e.g.*, UGUAA in NUE) was found in FUE, which was also shown in other studies (Loke *et al.* 2005; Shen *et al.* 2008a).

### Poly(A) sites in different genic and intergenic regions

It was demonstrated that APA sites located in different regions of the gene body may use different kinds of poly(A) signals (Wu *et al.* 2011). To understand the variations of polyadenylation, the single-nucleotide profiles and poly(A) signals were investigated with PAC data from the three different sequencing platforms as well as the combined one.

As shown in Figure 1, 5′-UTRs always have similar proportion of unique poly(A) sites in all four PAC datasets including ESTs (0.89%, Figure 1C), 454 (1.77%, Figure 1D), Illumina (1.51%, Figure 1E), and combined data (1.61%, Figure 1F). In Figure S4, it can be seen that NUE regions (−28 to −5) of 5′-UTR poly(A) sites have a clear U-peak followed by an A-peak, and the peaks are obvious in EST and 454 data (>40%), whereas Illumina data do not show such high peaks (~33%) in comparison with EST and 454 data. Poly(A) sites (−1 position) always possess a high A-peak (~50%) in all three datasets. In term of poly(A) signals, UGUAA is the most frequent signal in the NUE regions (Figure S4 and Table S1). UGUAA has an obviously greater frequency than other pentamers, especially in EST and 454 data. Because the collected poly(A) site number is too low in 5′-UTRs (~1%), RSAT cannot give the Z-score for the signals from all three datasets.

3′-UTRs always have the greatest poly(A) site numbers (*i.e.*, 38.44% in Illumina and >67% in EST and 454 PAC datasets, see Figure 1) in all the genic and intergenic regions. From the single nucleotide profiles (Figure S5), there are clearly an U-peak (>32%) following by an A-peak (>34%) in their NUE regions and a high A-peak (>49%) at poly(A) sites in all three datasets. In the NUE regions, the canonical pentamer UGUAA shows the greatest frequency (>24%) and significance (Z-score > 13 with order-3 Markov model) in all three datasets (Table S1). Other UGUAA-like pentamers (*e.g.*, CUGUA and UGCAA) are also detected with relatively high frequencies (>7%) and significance (Z-score > 9).

The frequency of PACs in CDS varies dramatically from 0.46% (ESTs) to 8.77% (Illumina) (Figure 1). Different from 5′-UTRs and 3′-UTRs, the single-nucleotide profiles in CDS have no clear U-peak and A-peak detected in the relevant NUE regions (−28 to −5) in all three datasets; and at poly(A) sites (−1 position) the A-peaks are only found in 454 and Illumina datasets (Figure S6). For the pentamers in the NUE region (Figure S6 and Table S1), UGUAA still has the greatest frequency (19.61%) in ESTs (Z-score cannot be given by RSAT because of small dataset). Yet in the 454 dataset, UGUAA still can be found but with low frequency (5.50%). The Illumina dataset does not have UGUAA in the top 50 ranked pentamers but other signals (*e.g.*, GGCGG and GCGGG) are found in the NUE region (Figure S6 and Table S1).

ESTs and 454 have similarly low percentage (0.66% and 1.23%) of poly(A) sites located in introns, yet there are more poly(A) sites detected in introns in Illumina data (14.98%) (Figure 1). The single-nucleotide profiles and signals in the NUE regions of intronic poly(A) sites are also similar between EST and 454 datasets, there is a clear U-peak followed by an A-peak and UGUAA (~35%) is dominant in NUE regions (Figure S7). In Illumina dataset, however, the single-nucleotide profile has dramatically high G content (47.53% in average) and no clear U-peak and A-peak is detected; UGUAA is not detected but G-rich pentamers (*e.g.*, GGUGG) are found with high frequencies (47.80%) and significance (Z-score = 22.33) in the NUE regions of intronic poly(A) sites (Table S1).

The percentages of PACs in intergenic regions increase from ESTs, 454 to Illumina (13.84%, 27.18%, and 36.31%, respectively, Figure 1). For intergenic poly(A) sites, the single-nucleotide profiles and pentamer signals are similar in ESTs and 454; an U-peak is followed by an A-peak in the NUE regions and a dominant A-peak is also found at poly(A) site (−1 position); UGUAA has the highest frequency (34.05% and 21.12%) and significance (Z-score = 4.98 and 10.46) in these two datasets (Figure S8, A and B). In Illumina data, the single nucleotide profile always has high G content (33.17% in average) except at poly(A) sites (Figure S8C), which is similar to those intronic poly(A) sites (Figure S7C). There is a weak U-peak (25.84%) followed by an A-peak (23.63%) in the NUE region, and UGUAA is not dominant (8.57% in frequency) but G-rich signals (*e.g.*, GUGGG, GGUGG, and GAGGG) are found with high significance (Z-score > 9) (Table S1).

Our results imply that different platforms (with different sequencing depths and coverage) might pick up different groups of transcripts that vary in poly(A) site selection. Although independent confirmations are needed, *e.g.*, authenticity of the poly(A) sites identified in each platform, one would assume deeper sequencing approach like Illumina might generate better coverage of poly(A) sites.

### Introns and CDS with PACs are larger in sizes

**Figure 3:**
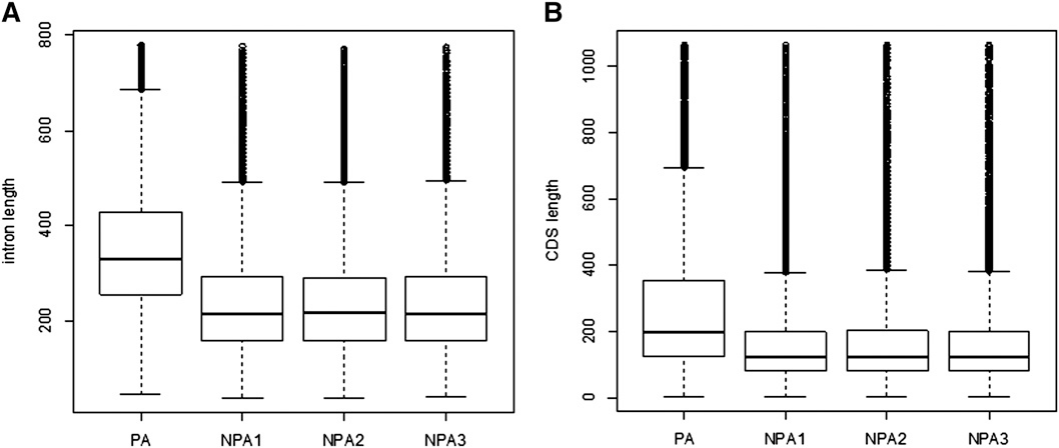
The length difference of intron and coding sequences (CDS) with and without poly(A) site clusters. (A) Intron. (B) CDS. PA: poly(A) sites; NPA1: control group 1 without poly(A) sites; NPA2: control group 2 without poly(A) sites; NPA3: control group 3 without poly(A) sites.

Previous reports indicated that poly(A) sites tend to be located in longer introns in plants (Wu *et al.* 2011) and animals (Tian *et al.* 2007). With available larger datasets, we were able to analyze the similar issue in *C. reinhardtii*. Indeed, we found that the introns with PACs are much longer than those without an internal PAC. There were 13,476 PACs located in 11,780 introns from 5,764 genes. Three control groups (NPA1, NPA2 and NPA3) with the same intron counts (11,780) but without a PAC were randomly selected as control datasets. We found that the average length of introns with and without PACs are 451 nt (median = 345) and 258 nt (median = 217), respectively. Apparently, the mean length of introns with PACs are significantly larger than those without PACs (Figure 3A, Wilcoxon tests, p-value < 2.2e-16).

Similarly, the CDS regions with PACs are also longer than those without a PAC. In our data analysis, 8102 PACs were found in 6953 CDS regions of 4937 genes. Similarly to the analysis in introns, in three randomly selected control groups of CDS regions (without PACs), the average length of CDS with and without PACs are 513 nt (median = 224) and 235 nt (median = 128), respectively. Statistically, the sizes of CDS with PACs are significantly larger than those without PACs (Figure 3B, Wilcoxon tests, p-value < 2.2e-16). Our results suggest a similar overarching phenomenon where longer introns and CDSs are better targets for the polyadenylation machinery.

### The preference of polyadenylation on spliced genes in *C. reinhardtii*

Identification of a large number of intronic poly(A) sites allowed us to examine the distribution of them in different kinds of introns: constitutive (spliced in all transcripts) and retained introns (not spliced in some transcripts). There are 13.82% (13,476) PACs located in intron regions in the combined dataset (Figure 1F), such sites are referred to as intronic PACs. Intronic PACs based on the categories such as constitutive/retained introns from protein-coding and non-coding genes are shown in Table 2. There were 139,859 (=134,708 + 5151) introns from coding genes and 2666 (=2550 + 116) introns from potential noncoding genes. Intronic PACs are almost equal percentage-wise between the introns of coding and noncoding genes (compare mean value of 5.63% and 5.16%, respectively) in average, although constitutively spliced introns are over two times of retained introns (compare mean value of 7.47% over 3.32%, respectively) in average. The results may indicate that polyadenylation machinery in *C. reinhardtii* has no preference on introns of coding over noncoding genes, but strongly prefers constitutive than retained introns, influencing the selection of splicing sites and gene expression in RNA posttrancriptional regulation.

### Association of poly(A) signal UGUAA usage with transcript levels

To further investigate the potential function of APA in gene expression regulation, we examined the relationship between the level of transcription and the use of particular poly(A) sites. Based on the definition of four different poly(A) site categories (*i.e.*, constitutive, strong, median and weak poly(A) sites, see the section *Materials and Methods* for definition), the numbers and percentages of the four different PAC sites and corresponding genes are shown in Table 3. In comparison with median poly(A) sites, there are less constitutive and strong poly(A) sites [2747 (4.93%) and 3653 (6.56%), respectively], and the corresponding genes are also less [2747 (16.05%) and 3653 (21.35%), respectively]. Because weak poly(A) sites are located in the same genes with strong sites, so it has the same gene number (3653, 21.35%) with strong sites. Most PACs are median poly(A) sites (39,044, 70.07%) and nearly half of all annotated genes (7946, 46.43%) possess median sites.

**Table 3:** Number of PACs in different categories

Poly(A) Site classification	No. of PAC	PACs Percentage	No. of Genes	Gene Percentage
Constitutive	2747	4.93	2,747	16.05
Strong	3653	6.56	3,653	21.35
Weak	10,276	18.44	3,653	21.35
Median	39,044	70.07	7,946	46.43

PAC, poly(A) site clusters.

**Figure 4:**
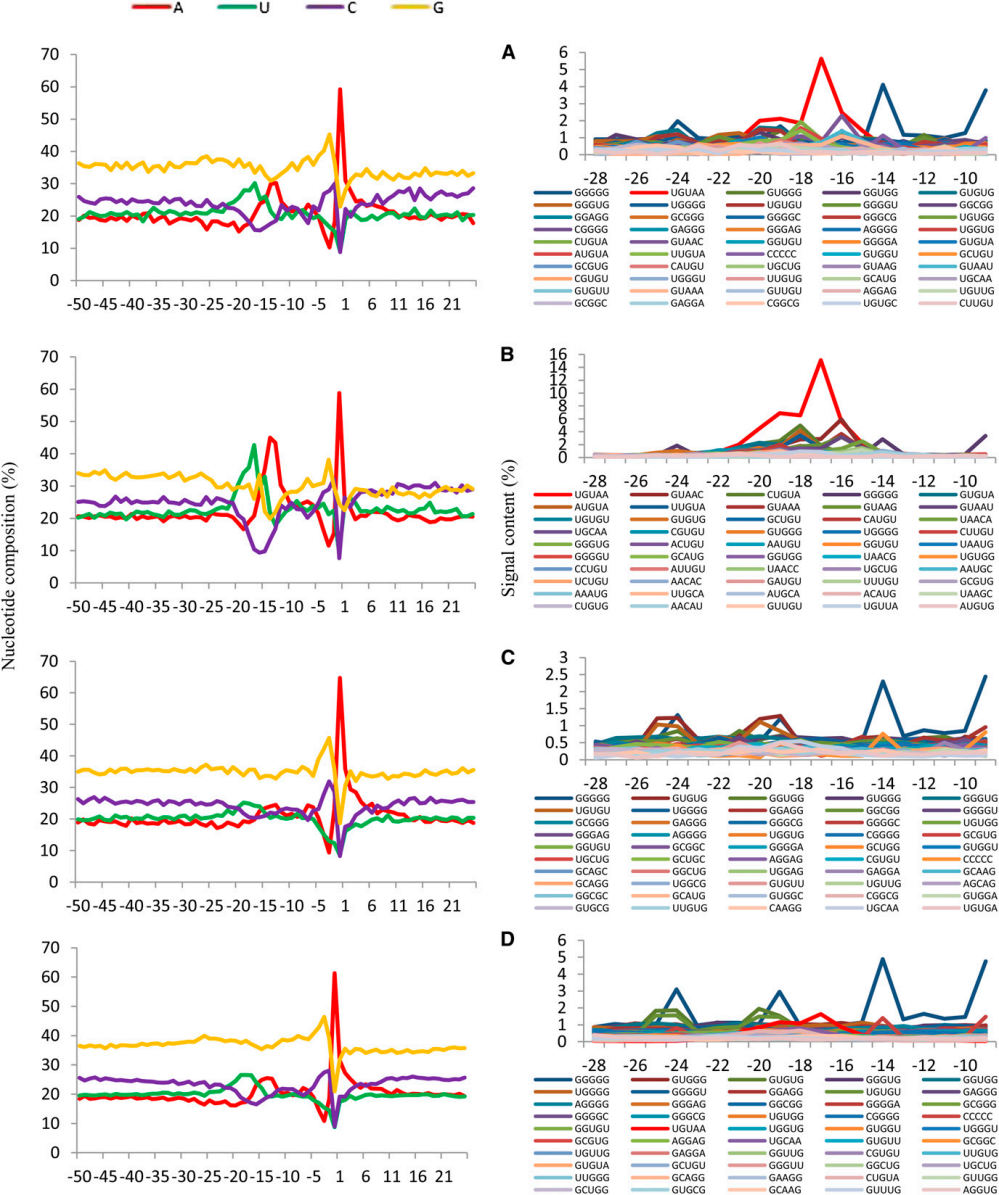
The single nucleotide profiles (−50 to +25) and putative poly(A) signals in near upstream element (NUE) region (−28 to −5) from different poly(A) site categories. (A) Constitutive poly(A) sites. (B) Strong poly(A) sites. (C) Weak poly(A) sites. (D) Median poly(A) sites.

To study whether different site categories have distinct nucleotide composition and poly(A) signals, the single nucleotide profiles (−50 to +25) and signals in the NUE regions (−28 to −5) are shown in Figure 4. It is obvious that strong poly(A) sites have high U-peak (42.70%) and A-peak (45.00%) in the NUE region and dominant A-peak at poly(A) sites in the single nucleotide profile, and UGUAA has the highest frequency (46.45%) and significance (Z-score = 8.04 with order-3 Markov model) among the pentamers of the NUE region (Figure 4B). Then constitutive poly(A) sites have similar features as strong sites but with high G-content (34.67% in average) in the single nucleotide profile and G-rich signals in the NUE region (Figure 4A). Both weak and median poly(A) sites have similar features: high G-content (34.89% and 36.62% respectively) in the single nucleotide profiles and low UGUAA frequencies, especially in weak sites where UGUAA is not detected in the top 50 frequent pentamers (Figure 4, C and D). This result is indicative of the clear association of the best poly(A) signal UGUAA and transcript levels, another evident reflecting the significance of the dominant signal in *C. reinhardtii*.

### APA extent variation among different datasets

**Figure 5:**
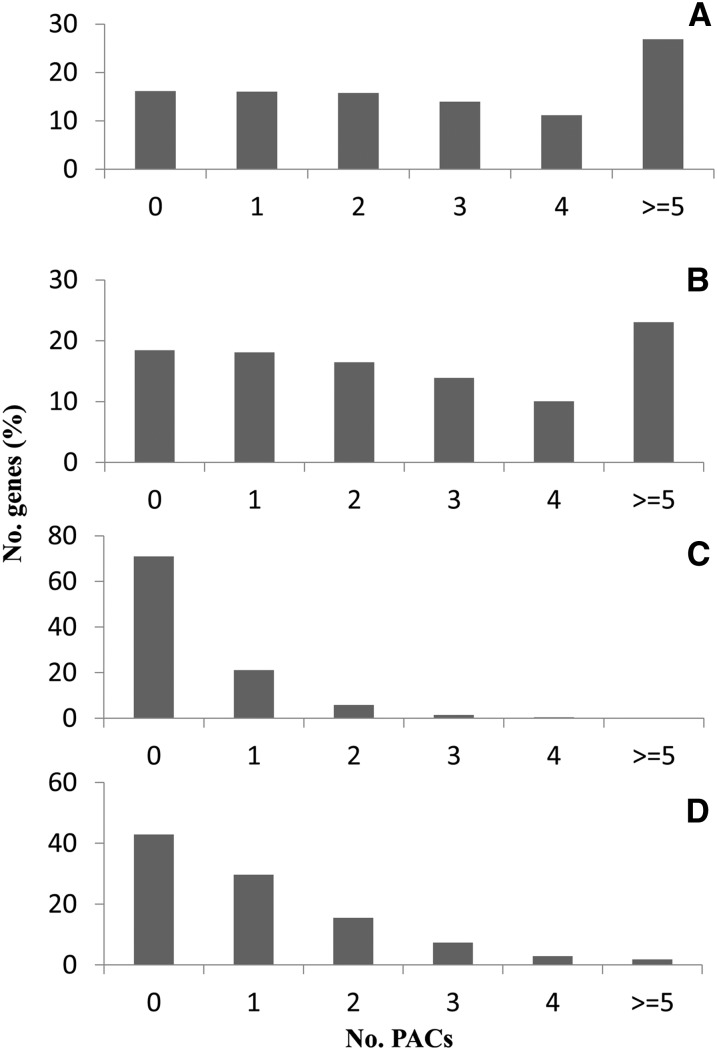
The gene numbers with different PACs among different datasets. (A) All poly(A) data (including Sanger expressed sequence tags (ESTs), 454, and Illumina). (B) Illumina data. (C) EST data. (D) 454 data. PACs, Poly(A) site clusters.

To examine whether different sequencing platforms generated different number of APA events, we tallied the number of genes with PACs from different datasets (Figure 5). The APA extent varies dramatically based on different datasets (Table 4). It was found that the APA extent was up to 67.78% of genes according to the total PACs in *C. reinhardtii*. Yet only 7.87% of genes had APA based on the EST data; following the increase of datasets, the APA extent was dramatically increased up to 63.46% in the Illumina dataset. Statistical analysis showed that there was significant relationship between APA extent and the size of poly(A) datasets (Pearson correlation coefficient: *r* = 0.995, *P* = 0.005). Clearly, there were less genes with multiple PACs, especially for high quality group (≥5 PACs), in small datasets. For example, there were only 0.11% and 1.80% genes with at least five PACs based on 6770 and 17,828 PACs in EST and 454 datasets, respectively (Figure 5, C and D). In Illumina dataset, in contrast, 23.05% genes had at least five PACs, considering that there are 50,664 PACs in total.

**Table 4:** APA extent variation among the four datasets

Dataset	No. PACs	APA Extent, %
EST	11,035	7.87
454	30,086	27.49
Illumina	88,304	63.46
Total	97,479	67.78

APA, alternative polyadenylation; PACs, poly(A) site clusters.

It is of great interest for us to answer the question on the potential biological functions of APA, particularly for those genes rich in APA (*i.e.*, the genes with at least five PACs). We performed GO analysis using the GO annotation v4.0 from JGI and GOEAST (Zheng and Wang 2008). The results of the highly significant GO functions are listed in Table 5. The result shows that the significant GO terms involve molecular functions and biological processes, mainly including receptor activities, noncoding RNA processing, protein synthesis, hydrolase, and ligase activities. As shown in Table 6, we also conducted GO analysis for those genes without any PAC. In comparison with Table 5, it is obviously that non-PAC genes mainly involve in amino acid activation and protein synthesis process, whereas high-quality APA genes could be related with RNA regulation, RNA processing, and signal response (receptors), among others. Further research on these gene categories may reveal detailed functionalities of these APA events.

**Table 5:** The most significant GO functions in high-quality APA genes with five or more PACs

GO_ID	Ontology	Term	P value
GO:0004872	molecular_function	Receptor activity	3.24E-33
GO:0034660	biological_process	ncRNA metabolic process	1.16E-23
GO:0008236	molecular_function	Serine-type peptidase activity	3.95E-23
GO:0017171	molecular_function	Serine hydrolase activity	3.95E-23
GO:1901135	biological_process	Carbohydrate derivative metabolic process	5.48E-23
GO:0006399	biological_process	tRNA metabolic process	1.07E-21
GO:0006418	biological_process	tRNA aminoacylation for protein translation	1.34E-18
GO:0043038	biological_process	Amino acid activation	1.34E-18
GO:0043039	biological_process	tRNA aminoacylation	1.34E-18
GO:0009056	biological_process	Catabolic process	1.43E-17
GO:0004812	molecular_function	Aminoacyl-tRNA ligase activity	5.44E-16
GO:0016875	molecular_function	Ligase activity, forming carbon-oxygen bonds	5.44E-16
GO:0016876	molecular_function	Ligase activity, forming aminoacyl-tRNA and related compounds	5.44E-16
GO:0003824	molecular_function	Catalytic activity	8.21E-16
GO:0016798	molecular_function	Hydrolase activity, acting on glycosyl bonds	2.26E-12
GO:0004553	molecular_function	Hydrolase activity, hydrolyzing O-glycosyl compounds	2.77E-12

GO, Gene Ontology; APA, alternative polyadenylation; PACs, poly(A) site clusters; ncRNA, noncoding RNA; tRNA, transfer RNA.

**Table 6:** The most significant GO functions in non-PAC genes

GO_ID	Ontology	Term	P value
GO:0043038	biological_process	Amino acid activation	6.580e-25
GO:0043039	biological_process	tRNA aminoacylation	6.580e-25
GO:0006418	biological_process	tRNA aminoacylation for protein translation	6.580e-25
GO:0004812	molecular_function	Aminoacyl-tRNA ligase activity	7.184e-24
GO:0016875	molecular_function	Ligase activity, forming carbon-oxygen bonds	7.184e-24
GO:0016876	molecular_function	Ligase activity, forming aminoacyl-tRNA and related compounds	7.184e-24
GO:0005044	molecular_function	Scavenger receptor activity	2.591e-21
GO:0038024	molecular_function	Cargo receptor activity	2.591e-21
GO:0034660	biological_process	ncRNA metabolic process	1.836e-20
GO:0006399	biological_process	tRNA metabolic process	1.234e-18
GO:0008236	molecular_function	Serine-type peptidase activity	2.101e-13
GO:0017171	molecular_function	Serine hydrolase activity	2.101e-13
GO:0016070	biological_process	RNA metabolic process	2.223e-13
GO:0004872	molecular_function	Receptor activity	1.871e-12

GO, Gene Ontology; PACs, poly(A) site clusters; ncRNA, noncoding RNA; tRNA, transfer RNA.

## Discussion

Deep sequencing technologies provided us with unprecedented genome and transcriptome information. With a much larger collection of poly(A) site data in the model organism *C. reinhardtii*, we are able to perform an in-depth analysis of the distribution of polyadenylation sites, polyadenylation signals and their relationship with splicing and gene expression levels. Our results provide further information about this model algal organism in terms of its RNA processing profiles and the potentials of APA in regulating gene expression.

### Poly(A) site distribution and polyadenylation profile variation in the genome and different datasets

According to current genome annotation, there are ~38% of PACs located in intergenic regions. This may be a result of inaccurate or incomplete annotation from insufficient numbers of ESTs/cDNA and reads used for the gene annotation. After expanding the 3′-end of genome annotation by 50 nt, the unique 3′-UTR poly(A) sites increase to 55.67%, whereas there still are 28.48% sites located within intergenic regions. These intergenic poly(A) sites might be from unannotated genes or indicate the presence of some novel polyadenylated transcripts (Lopez *et al.* 2006; Wu *et al.* 2011). Furthermore, >500 bp extension (relative to reference 3′-UTRs) was identified in long intergenic noncoding RNAs in mouse and human brain tissues (Miura *et al.* 2013). Such notable difference may be due to the biological differences between green algae and mammals (*e.g.*, mRNA abundance and gene compactness).

From poly(A) site distribution, which is similar to the results of Shen *et al.* (2008a) in which they used 16,952 *in silico*-verified poly(A) sites from EST sequences (Shen *et al.* 2008a), it is shown that ESTs have much more similar distribution features to 454 than Illumina in the whole genome among the three datasets from different sequencing platforms (Figure 1). This may be caused by the difference of deep-sequencing reads considering that the reads in 454 are much longer (>400 bp) than Illumina (~50 bp). Moreover, deep-sequencing (454 and Illumina) can reveal much more poly(A) sites than Sanger sequencing. Interestingly, 5′-UTRs always have low site abundance (~1%) across the three datasets, which may suggest that 5′-UTRs are more stable in transcription and/or translation and may be less involved in APA events. Such result is consistent with the hypothesis that shorter pre-AUG poly(A) tract in 5′-UTRs can bind to translation initiation factors to enhance translation initiation, while a long tract (≥12) will bind to Pab1p (a poly(A) binding protein) resulting in repression of translation in yeast (Xia *et al.* 2011).

The variations of single nucleotide profiles and poly(A) signals are clearly different among different genic and intergenic regions. 3′-UTRs have the most dominant UGUAA signal, which is also consistent with the previous papers (Wodniok *et al.* 2007; Shen *et al.* 2008a) in *C. reinhardtii*. No clear NUE region or significant poly(A) signal is detected in CDS poly(A) sites in all the three datasets (Figure S6), and similar result also was found in Arabidopsis (Wu *et al.* 2011). In Illumina data, the single-nucleotide profiles and signals in intron and intergenic regions have much greater G content than those in EST and 454 data, and such feature could be caused by extremely biased base composition in Illumina sequencing (Oyola *et al.* 2012), because the genome of *C. reinhardtii* is strongly GC-biased considering that the GC content is up to 63.45%, which is significantly greater than other multicellular organisms (Merchant *et al.* 2007). Through DRS technology, novel poly(A) signals TTTTTTTTT and AAWAAA (W = A/T) were found around NUE regions in human (Ozsolak *et al.* 2010). Such results may be also caused by sequencing bias because human genome is AT-rich. Another possible explanation is internal priming, and it has been reported that internal priming would cause artifacts within protein-coding regions by reverse transcriptase when using an oligo(dT)-based primers in Arabidopsis DRS sequencing (Sherstnev *et al.* 2012). The different single nucleotide profiles and poly(A) signals among different genic regions in *C. reinhardtii* indicate that polyadenylation mechanism may be diversified in eukaryotes, and more deep-sequencing data (beyond 3′-UTRs) are needed to give further analysis in other genic regions beyond 3′-UTRs.

### The influence of polyadenylation on splicing events in *C. reinhardtii*

In our study, CDS and introns with poly(A) sites are longer than those without poly(A) sites, and similar results are also found in intronic poly(A) sites in Arabidopsis (Wu *et al.* 2011) and human (Tian *et al.* 2007). Although a weak 5′ splice site (5′ss) is detected in introns with poly(A) sites in Arabidopsis (Wu *et al.* 2011) and human (Tian *et al.* 2007), no significant difference in the strength of 5′ ss signal was detected between the introns with poly(A) sites and without poly(A) sites in *C. reinhardtii* in our study (data not shown). Therefore, it seems that polyadenylation may not prefer weak 5′ss, and UGUAA-dominated species (*e.g.*, *C. reinhardtii*) may have distinct splice site selection in polyadenylation from AAUAAA-dominated species (*e.g.*, Arabidopsis and human).

Although intron retention made up half of the alternative splicing events (305 of 611) in *C. reinhardtii* (Labadorf *et al.* 2010), our study shows that constitutively spliced introns are over two times more enriched with poly(A) sites than that of retained introns, suggesting constitutively spliced introns have stronger preference to polyadenylation than retained introns. So it is possible that the preference of polyadenylation to constitutive splicing may be the result of interplay between polyadenylation and splicing. Another explanation could be that constitutively spliced introns are more likely than retained introns to contain noncoding RNAs, which are stabilized by polyadenylation after excision by splicing.

### Alternative polyadenylation in *C. reinhardtii*

It was found that the extent of APA in *C. reinhardtii* is up to 68% based on our combined data. We used a stringent criterion (each site with at least three ESTs/reads support) and new annotation file (v4.3) to filter the unique poly(A) site and obtain PACs that can appropriately define high-quality APA events. Interestingly, the APA is only found in 8% of genes based on our EST data (from 21,041 unique poly(A) sites); whereas Shen *et al.* (2008a) reported up to 33% APA based on 16,952 poly(A) sites from EST data and genome annotation v3.1. It is clear that the extent of APA depends on the size of dataset and accuracy of genome annotation, and similar results were also obtained in Arabidposis and rice through deep-sequencing (Wu *et al.* 2011; Shen *et al.* 2011).

In our study, more poly(A) sites were detected in CDS and intron regions, especially in deep-sequencing data (454 and Illumina); and there was no clear NUE region and significant poly(A) signal in CDS poly(A) sites. As known, APA located in intron/CDS regions *vs.* 3′-UTRs would result in dramatically different mRNA isoforms. APAs from intron/CDS and 3′-UTR may involve in different regulation mechanisms (Di Giammartino *et al.* 2011; Thomas *et al.* 2012). It was mentioned that poly(A) tag sequencing results from protein-coding regions (CDS/intron regions) may be dubious because of internal priming (Sherstnev *et al.* 2012), so more poly(A) sites detected in CDS and intron regions, especially in Illumina data, may not reflect the real number of poly(A) sites. It is equally possible that the differences of poly(A) signal in CDS/intron and 3′-UTR relate to different APA mechanisms (Wu *et al.* 2011; Thomas *et al.* 2012).

Although functions and potential mechanisms of APA have been reported in plants and animals (Ozsolak *et al.* 2009; Singh *et al.* 2009; Thomas *et al.* 2010; Mangone *et al.* 2010; Ozsolak *et al.* 2010; Xing and Li 2011; Wu *et al.* 2011; Shen *et al.* 2011; Di Giammartino *et al.* 2011), the verified biological functions and mechanism of APA genes in *C. reinhardtii* have not been reported. Our *in silico* analysis of APA genes indicates that receptor genes and noncoding RNA as well as protein metabolism are impacted by the APA in *C. reinhardtii*. However, non-PAC genes are less involved in RNA regulation but in protein synthesis process. The significance of these bioinformatics findings remains to be verified by wet-lab experiments in the near future. The results presented here would, without a doubt, benefit further research in the contribution of APA in gene expression of this and related organisms.

Polyadenylation is an essential step in eukaryotic mRNA posttranscriptional process, yet model organism green alga *C. reinhardtii* has an apparently different poly(A) signal (UGUAA) from most eukaryotes (*e.g.*, Arabidopsis and human). In this study, through genome-wide analysis from three different sequencing data (Sanger ESTs, 454, and Illumina), it is shown that deep-sequencing detected more new poly(A) sites, especially in CDS, intron, and intergenic regions; moreover, Illumina data revealed overexpressed G content around poly(A) sites, which may indicate unknown feature in polyadenylation or be contaminated in sequencing. The prevalence of different poly(A) signals between CDS and 3′-UTR implies potentially different mechanisms of polyadenylation. Our data suggest that the APA occurs in approximately 68% of *C. reinhardtii* genes. Moreover, intronic poly(A) sites are more abundant in constitutively spliced introns than retained introns, suggesting an interplay between polyadenylation and splicing. Our results support that APA, as in higher eukaryotes, may play significant roles in increasing transcriptome diversity and gene expression regulation in this green alga species. Our datasets also provide information for accurate annotation of transcript ends in the genome level.

## Supplementary Material

Supporting Information
