# Supplementary material for: Bioinformatics Analysis of Alternative Polyadenylation in Green Alga Chlamydomonas reinhardtii Using Transcriptome Sequences from Three Different Sequencing Platforms
Source: G3 (Bethesda). 2014 Mar 13;4(5):871–83. doi: 10.1534/g3.114.010249 (PMC4025486; doi:10.1534/g3.114.010249)
Supplement: Supporting Information [file supp_4_5_871__index.html]

Bioinformatics Analysis of Alternative Polyadenylation in Green Alga Chlamydomonas reinhardtii Using Transcriptome Sequences from Three Different Sequencing Platforms — Supporting Information 

# Bioinformatics Analysis of Alternative Polyadenylation in Green Alga *Chlamydomonas reinhardtii* Using Transcriptome Sequences from Three Different Sequencing Platforms

## Supporting Information for Zhao *et al.*, 2014

**Files in this Data Supplement:**

- Supporting Information - Figures S1-S8, Tables S1-S2, and Files S1-S2 (PDF, 1 MB)
- Figure S1 - The distance distribution of intergenic unique poly(A) sites after 3'-UTRs. (PDF, 338 KB)
- Figure S2 - The top frequent motifs from different datasets in the NUE region. (PDF, 449 KB)
- Figure S3 - The top frequent motifs from different datasets in the FUE region. (PDF, 649 KB)
- Figure S4 - The single nucleotide profiles (-50 to +25) and top frequent motifs in NUE regions (-28 to -5) of poly(A) sites in 5'-UTRs from different PAC datasets. (PDF, 519 KB)
- Figure S5 - The single nucleotide profiles (-50 to +25) and top frequent motifs in NUE regions (-28 to -5) of poly(A) sites in 3'-UTRs from different PAC datasets. (PDF, 449 KB)
- Figure S6 - The single nucleotide profiles (-50 to +25) and top frequent motifs in NUE regions (-28 to -5) of poly(A) sites in CDS from different PAC datasets. (PDF, 607 KB)
- Figure S7 - The single nucleotide profiles (-50 to +25) and top frequent motifs in NUE regions (-28 to -5) of poly(A) sites in intron from different PAC datasets. (PDF, 534 KB)
- Figure S8 - The single nucleotide profiles (-50 to +25) and top frequent motifs in NUE regions (-28 to -5) of poly(A) sites in intergenic regions from different PAC datasets. (PDF, 468 KB)
- Table S1 - The conserved pentamers detected in the NUE regions in *C. reinhardtii*. (PDF, 126 KB)
- Table S2 - The conserved pentamers detected in the FUE regions in *C. reinhardtii*. (PDF, 125 KB)
- File S1 - The final PAC sequences used for our data analysis. (.zip, 15 MB)
- File S2 - The source code of SignalSleuth2. (.zip, 6 KB)
