# Supplementary material for: Bioinformatics Analysis of Alternative Polyadenylation in Green Alga Chlamydomonas reinhardtii Using Transcriptome Sequences from Three Different Sequencing Platforms
Source: G3 (Bethesda). 2014 Mar 13;4(5):871–83. doi: 10.1534/g3.114.010249 (PMC4025486; doi:10.1534/g3.114.010249)
Supplement: Supporting Information [file supp_g3.114.010249_010249SI.pdf]

G3·Genes | Genomes | Genetics

Bioinformatics analysis of alternative polyadenylation in green alga *Chlamydomonas reinhardtii* using transcriptome sequences from three different sequencing platforms

Zhixin Zhao[1], Xiaohui Wu[1, 2], Praveen Kumar Raj Kumar[1], Min Dong[1, 2], Guoli Ji[2], Qingshun Quinn Li[1,3,4*] and Chun Liang[1*]

[1] Department of Biology, Miami University, Oxford, Ohio 45056, USA
[2] Department of Automation, Xiamen University, Xiamen, 361005, China
[3] Key Laboratory of the Ministry of Education for Coastal and Wetland Ecosystems, and College of the Environment and Ecology, Xiamen University, Xiamen, Fujian, China 361102
[4] Rice Research Institute, Fujian Academy of Agricultural Sciences, Fuzhou, Fujian, China 350003

*Corresponding authors

Email addresses:
ZZ: zhaoz@miamioh.edu
XW: xhuister@xmu.edu.cn
PKRK: rajkump@miamioh.edu
MD: dongm3@miamioh.edu
GJ: glji@xmu.edu.cn
QQL: liq@miamioh.edu
CL: liangc@miamioh.edu

DOI: 10.1534/g3.114.010249

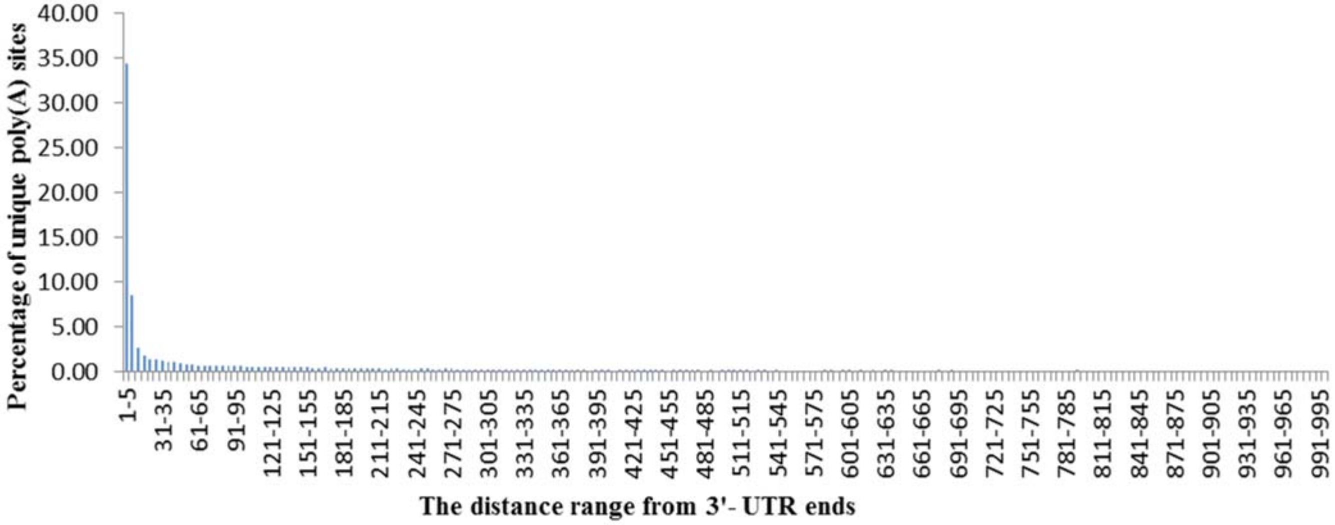

Figure S1 The distance distribution of intergenic unique poly(A) sites after 3'-UTRs. 1—1000 nt downstream of 3'-UTR is selected to investigate the distribution of poly(A) sites in intergenic region. X-axis shows the 200 sub-regions which are 5 nt in length (*e.g.*, 1—5 and 6—10). Y-axis labels the percentage of poly(A) sites for each sub-region.

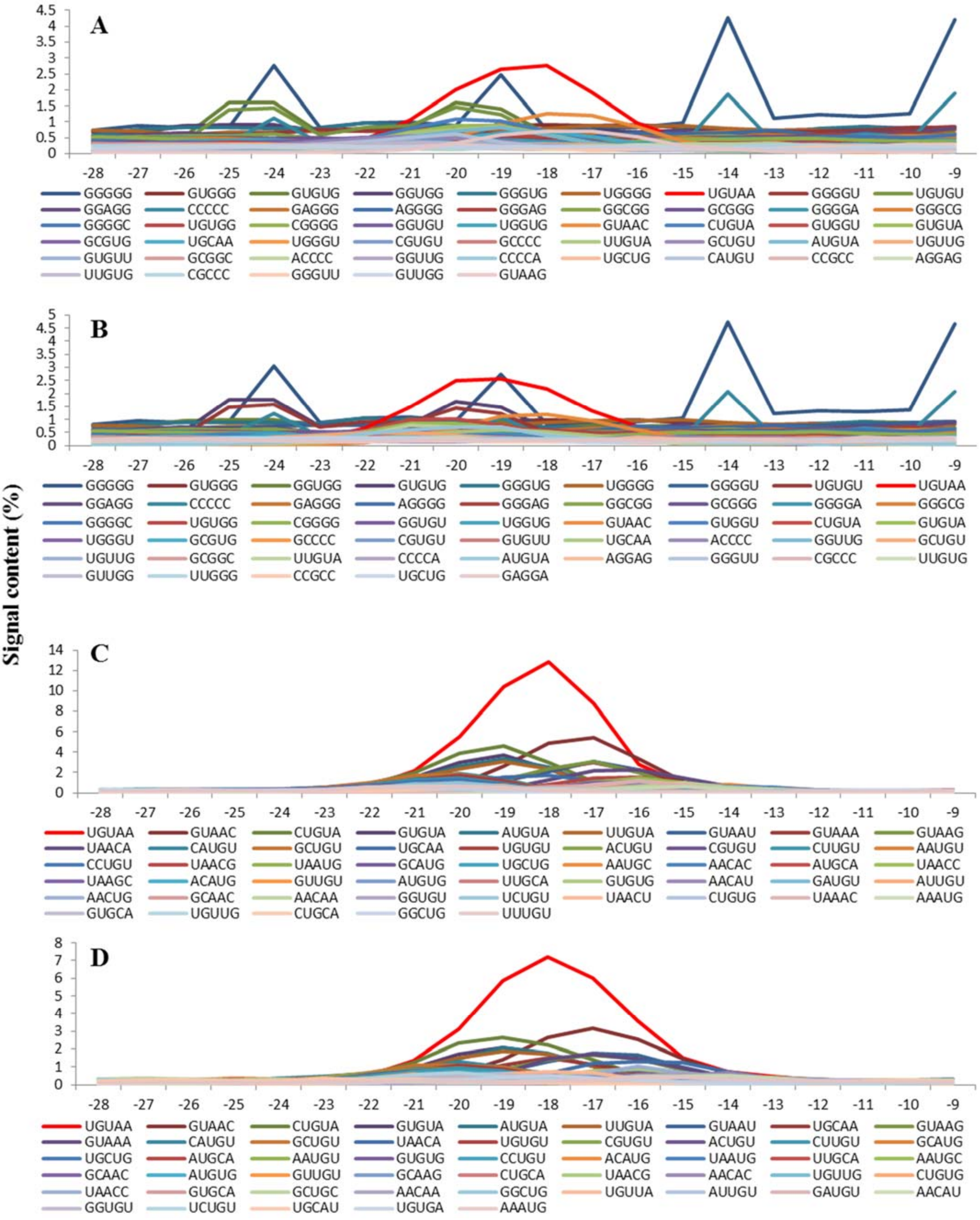

Figure S2 The top frequent motifs from different datasets in the NUE region. (A) All PAC data (including ESTs, 454 and Illumina). (B) Illumina data. (C) EST data. (D) 454 data.

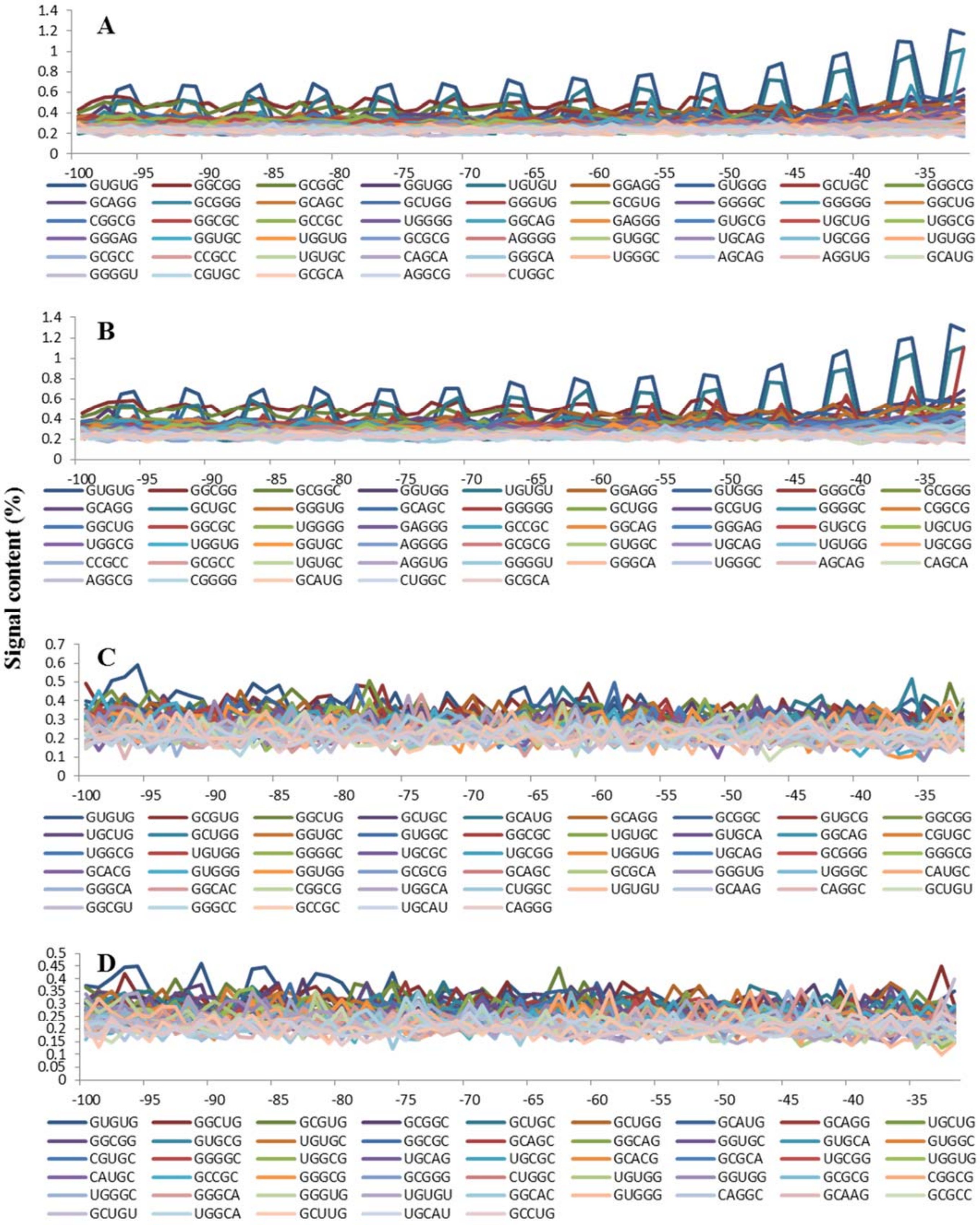

Figure S3 The top frequent motifs from different datasets in the FUE region. (A) All PAC data (including ESTs, 454 and Illumina). (B) Illumina data. (C) EST data. (D) 454 data.

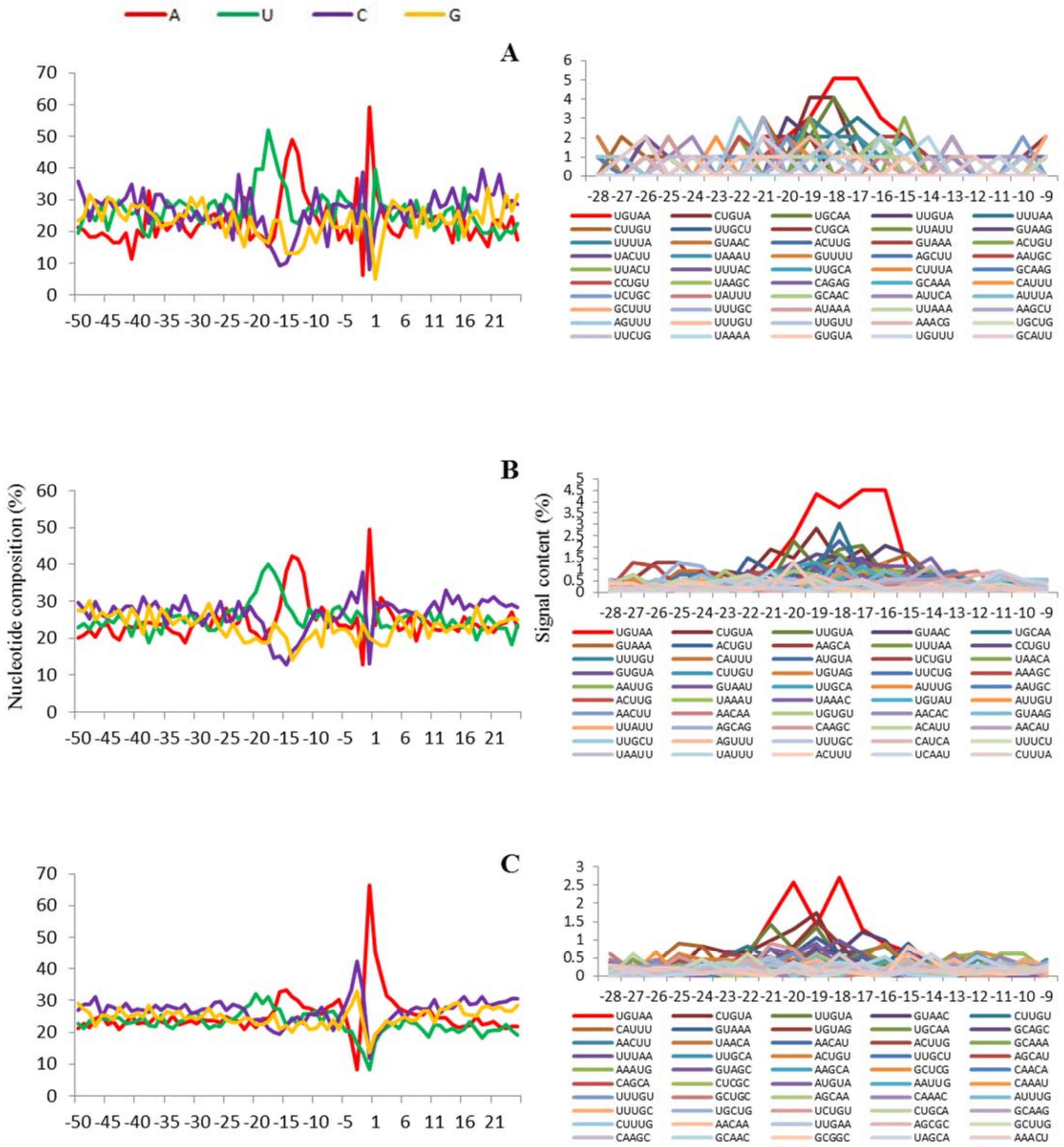

Figure S4 The single nucleotide profiles (-50 to +25) and top frequent motifs in NUE regions (-28 to -5) of poly(A) sites in 5'-UTRs from different PAC datasets. (A) EST data. (B) 454 data. (C) Illumina data.

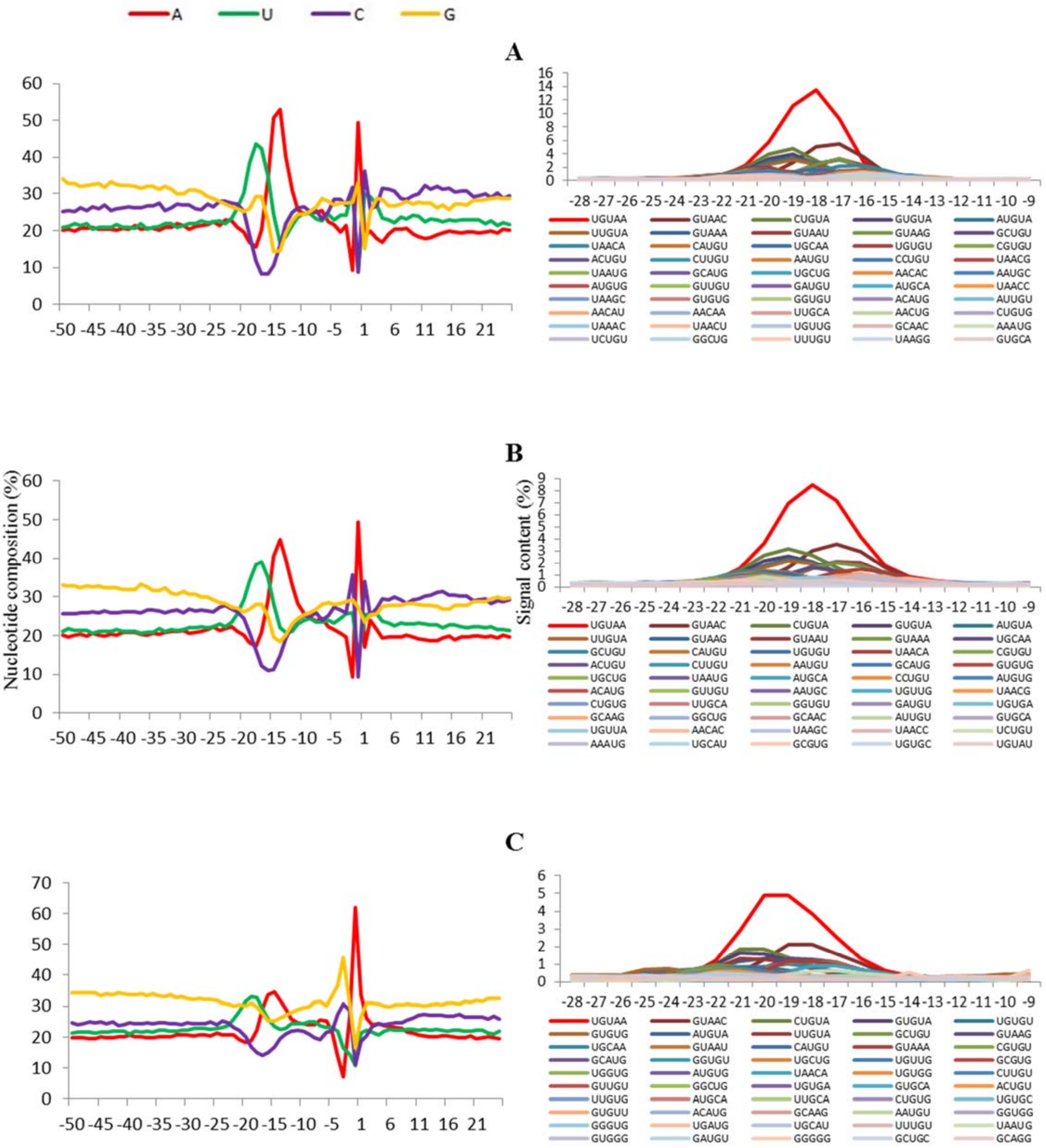

Figure S5 The single nucleotide profiles (-50 to +25) and top frequent motifs in NUE regions (-28 to -5) of poly(A) sites in 3'-UTRs from different PAC datasets. (A) EST data. (B) 454 data. (C) Illumina data.

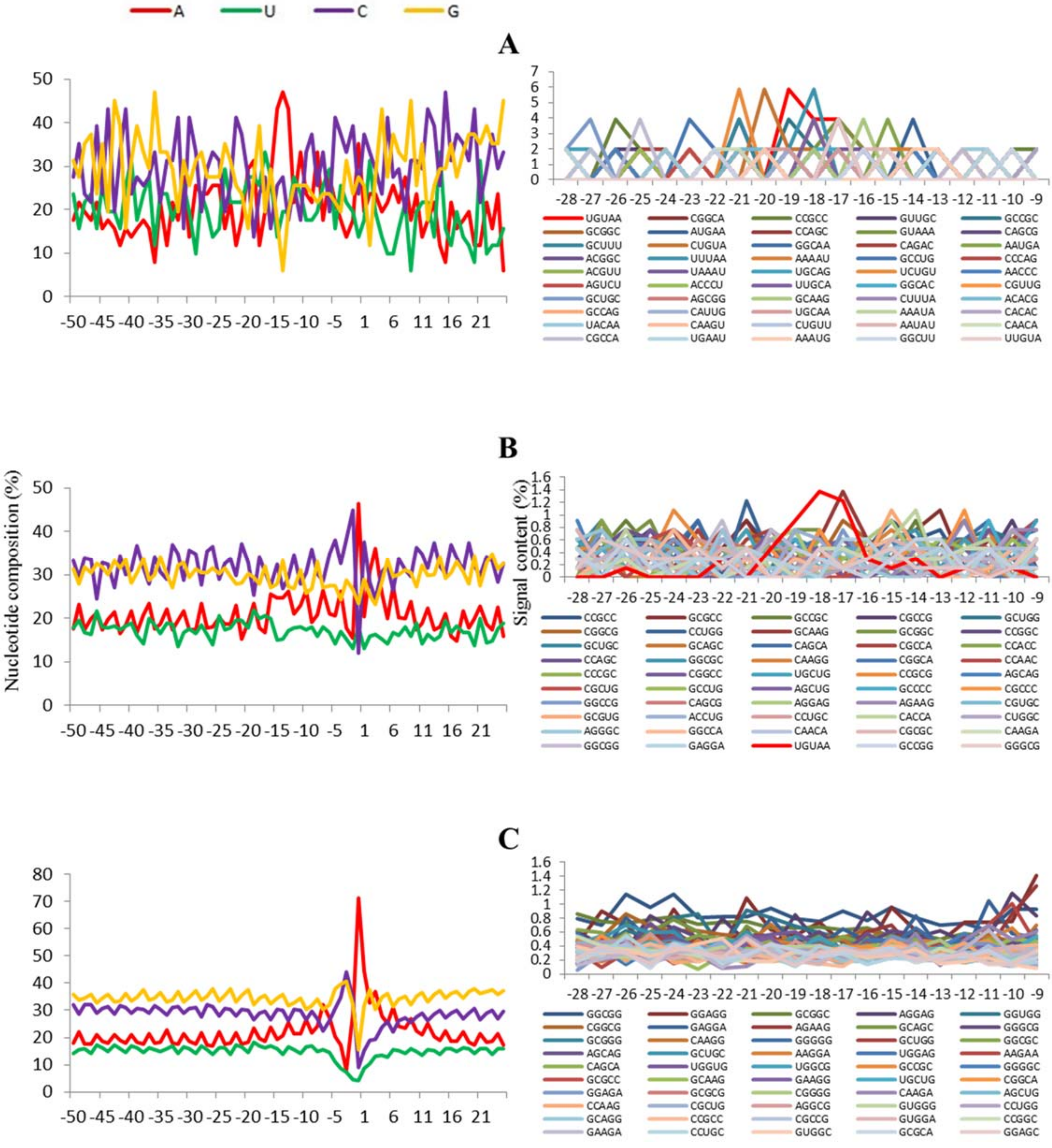

Figure S6 The single nucleotide profiles (-50 to +25) and top frequent motifs in NUE regions (-28 to -5) of poly(A) sites in CDS from different PAC datasets. (A) EST data. (B) 454 data. (C) Illumina data.

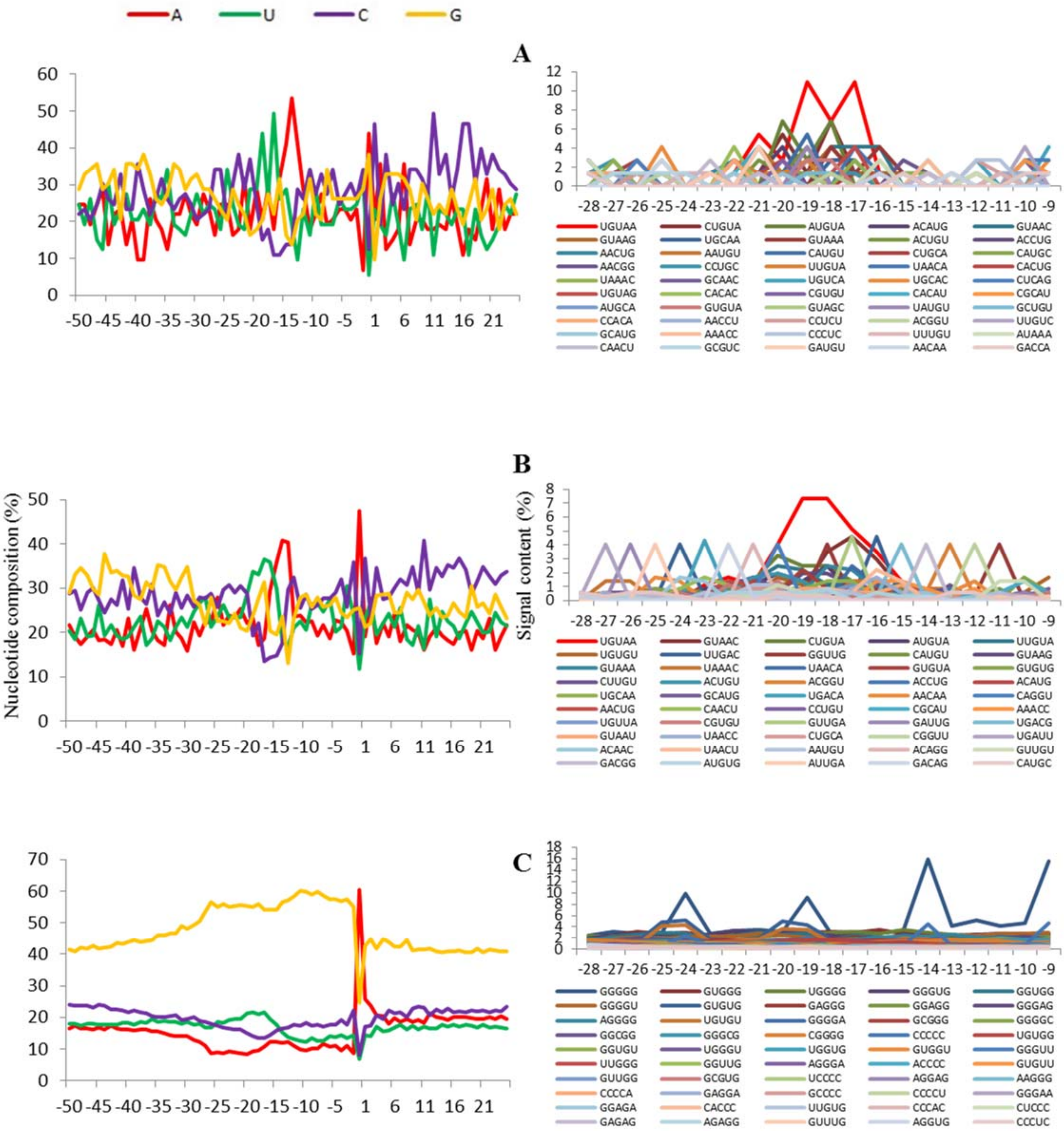

Figure S7 The single nucleotide profiles (-50 to +25) and top frequent motifs in NUE regions (-28 to -5) of poly(A) sites in intron from different PAC datasets. (A) EST data. (B) 454 data. (C) Illumina data.

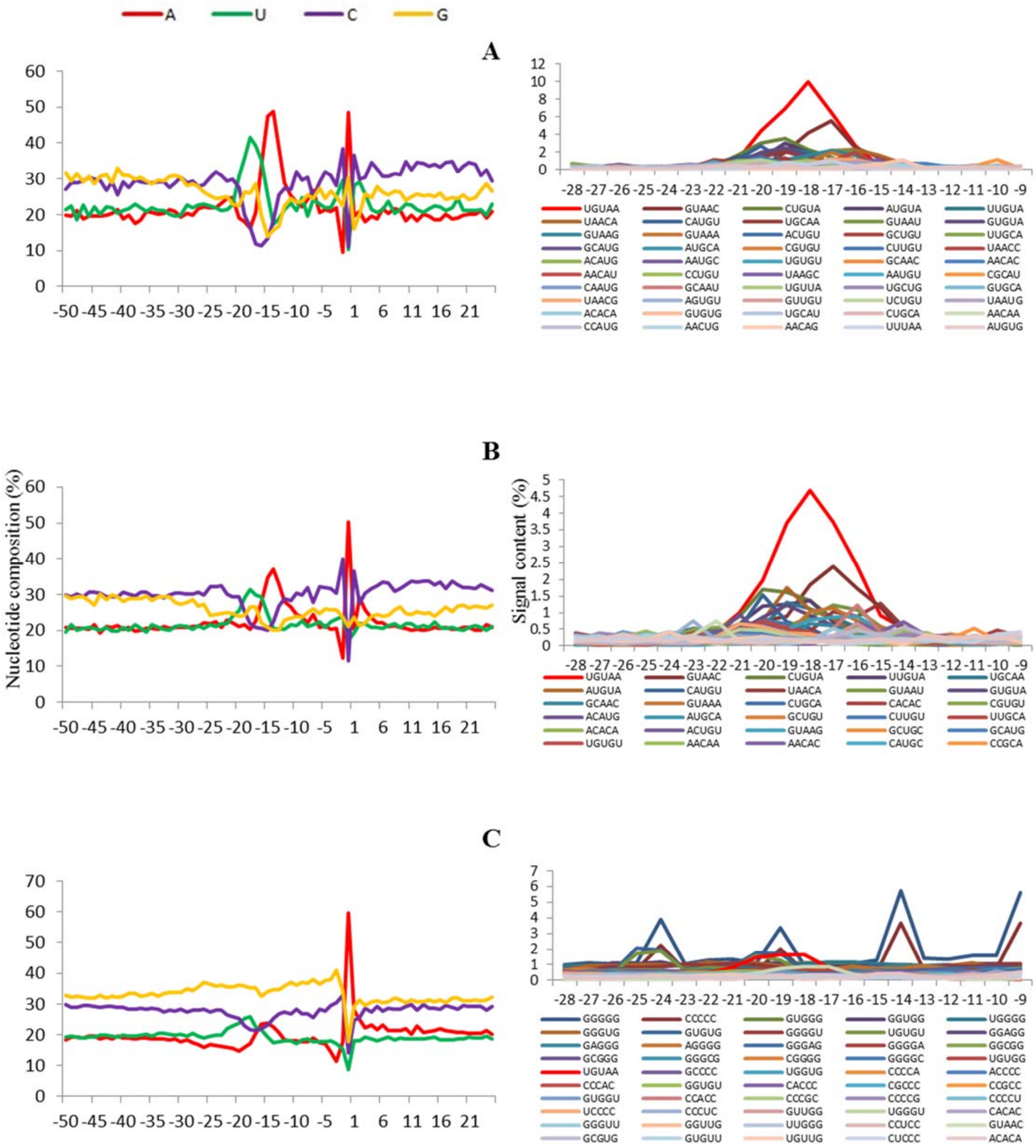

Figure S8 The single nucleotide profiles (-50 to +25) and top frequent motifs in NUE regions (-28 to -5) of poly(A) sites in intergenic regions from different PAC datasets. (A) EST data. (B) 454 data. (C) Illumina data.

Table S1 The conserved pentamers detected in the NUE regions in *C. reinhardtii*

Data	Category	Signal	Frequency (%)	Z-score with order-3 Markov model
All sites	Whole	UGUAA	13.39	32.88
		GUGGG	16.10	22.38
		GGUGG	15.16	39.61
		CUGUA	5.95	42.48
		UGCAA	5.00	23.16
Illumina	Whole	UGUAA	13.16	32.31
		GUGGG	17.63	21.02
		GGUGG	16.54	38.02
		GAGGG	11.70	18.02
		CUGUA	5.68	42.64
		UGCAA	4.67	21.36
	5'UTR	UGUAA	13.58	Data too small
	CDS	GGCGG	16.98	3.31
		GCGGG	8.45	3.88
	Intron	GGUGG	47.80	22.33
	3'UTR	UGUAA	24.38	25.87
		CUGUA	9.90	15.32
		UGCAA	7.32	16.93
	Intergenic	GUGGG	19.56	11.04
		GGUGG	18.69	23.50
		GAGGG	12.98	9.66
		GGGAG	12.36	8.82
		UGUAA	8.57	15.10
		GGCGG	10.97	11.03
		GCGGG	10.83	8.82
454	Whole	UGUAA	31.67	21.81
		GUAAC	14.14	10.24
		CUGUA	13.09	10.70
		UGCAA	8.37	16.93
	5'UTR	UGUAA	24.87	Data too small
	CDS	UGUAA	5.50	Data too small
	Intron	UGUAA	34.78	Data too small
	3'UTR	UGUAA	36.88	18.53
		CUGUA	14.96	9.53
		UGCAA	8.93	14.81
	Intergenic	UGUAA	21.12	10.46
ESTs	Whole	UGUAA	45.55	14.60
		GUAAC	19.78	7.43
		CUGUA	17.35	7.39
		UGCAA	8.72	11.12
	5'UTR	UGUAA	23.47	Data too small
	CDS	UGUAA	19.61	Data too small
	Intron	UGUAA	39.73	Data too small
	3'UTR	UGUAA	47.86	13.58
		GUAAC	20.36	6.71
		CUGUA	17.93	6.77
		UGCAA	8.57	10.45
	Intergenic	UGUAA	34.05	4.98

"Data too small" means Z-score cannot be calculated by RSAT because the input sequence number is too small.

Table S2 The conserved pentamers detected in the FUE regions in *C. reinhardtii*

Data	Signal	Frequency (%)	Z-score with order-3 Markov model
All sites	GUGUG	35.51	None
	GCGGC	28.90	None
	GGUGG	27.35	22.88
	GUGCG	19.93	17.50
	GUGGG	24.43	14.81
	CGUGC	15.73	14.60
	GCGUG	22.72	13.11
	UGUGU	27.19	12.38
	GGCGG	33.30	10.87
Illumina	GUGUG	37.25	None
	GCGGC	29.71	None
	GGUGG	28.75	27.29
	GUGCG	20.19	19.05
	GUGGG	25.59	16.20
	GCGUG	23.16	15.75
	GAGGG	21.27	15.26
454	GUGUG	21.91	None
	GCGUG	21.11	5.23
	GCAUG	19.69	4.14
	GUGCG	18.88	4.46
ESTs	GUGUG	23.87	None
	GCGUG	23.64	3.34
	GGGCC	14.83	3.52
	UGCAU	14.71	4.18

"None" means Z-score was too low to be on the listed generated by RSAT.

Files S1-S2

Available for download as .zip files at http://www.g3journal.org/lookup/suppl/doi:10.1534/g3.114.010249/-/DC1

File S1 The final PAC sequences used for our data analysis.

File S2 The source code of SignalSleuth2
