# Supplementary material for: Bioinformatics Analysis of Alternative Polyadenylation in Green Alga Chlamydomonas reinhardtii Using Transcriptome Sequences from Three Different Sequencing Platforms
Source: G3 (Bethesda). 2014 Mar 13;4(5):871–83. doi: 10.1534/g3.114.010249 (PMC4025486; doi:10.1534/g3.114.010249)
Supplement: Supporting Information [file supp_g3.114.010249_FigureS1.pdf]

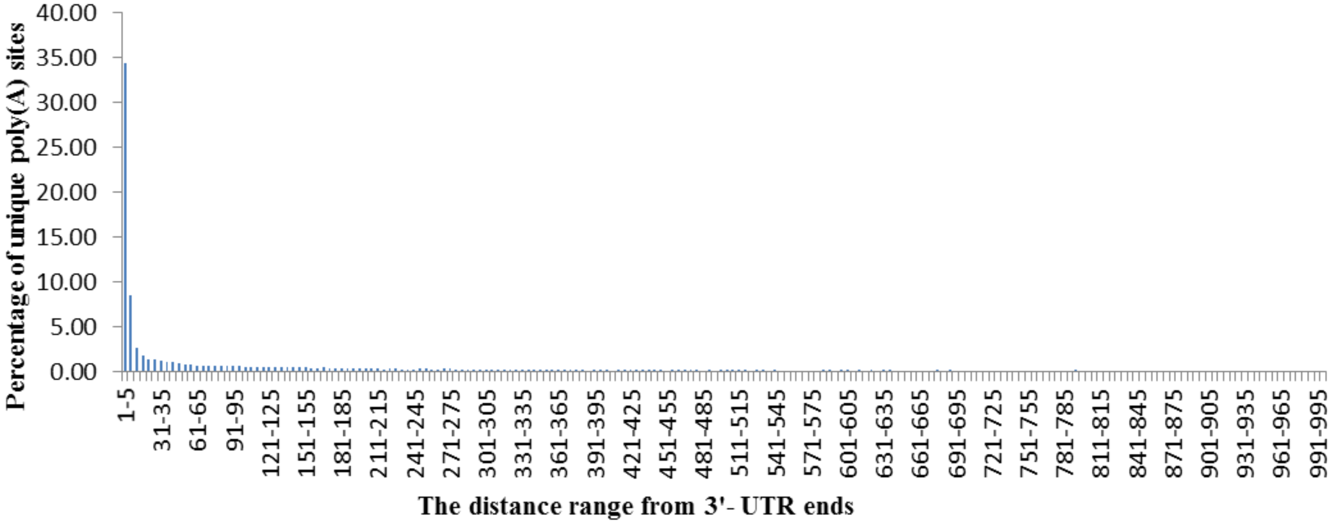

Figure S1 The distance distribution of intergenic unique poly(A) sites after 3'-UTRs. 1－1000 nt downstream of 3'-UTR is selected to investigate the distribution of poly(A) sites in intergenic region. X-axis shows the 200 sub-regions which are 5 nt in length (*e.g.*, 1－5 and 6－10). Y-axis labels the percentage of poly(A) sites for each sub-region.
