# Supplementary material for: Bioinformatics Analysis of Alternative Polyadenylation in Green Alga Chlamydomonas reinhardtii Using Transcriptome Sequences from Three Different Sequencing Platforms
Source: G3 (Bethesda). 2014 Mar 13;4(5):871–83. doi: 10.1534/g3.114.010249 (PMC4025486; doi:10.1534/g3.114.010249)
Supplement: Supporting Information [file supp_g3.114.010249_FigureS5.pdf]

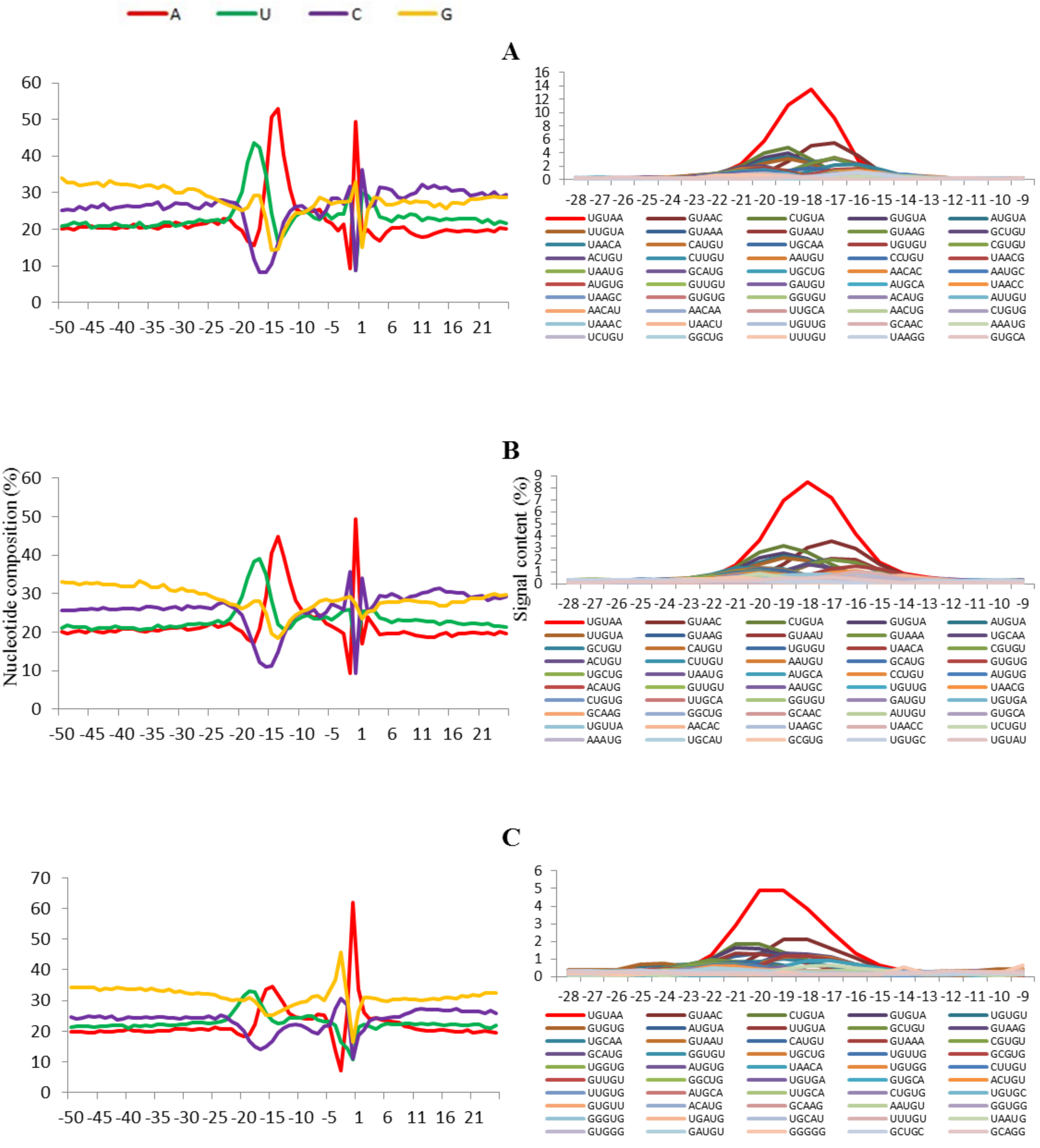

Figure S5 The single nucleotide profiles (-50 to +25) and top frequent motifs in NUE regions (-28 to -5) of poly(A) sites in 3'-UTRs from different PAC datasets. (A) EST data. (B) 454 data. (C) Illumina data.
