# Supplementary material for: Bioinformatics Analysis of Alternative Polyadenylation in Green Alga Chlamydomonas reinhardtii Using Transcriptome Sequences from Three Different Sequencing Platforms
Source: G3 (Bethesda). 2014 Mar 13;4(5):871–83. doi: 10.1534/g3.114.010249 (PMC4025486; doi:10.1534/g3.114.010249)
Supplement: Supporting Information [file supp_g3.114.010249_TableS1.pdf]

Table S1 The conserved pentamers detected in the NUE regions in *C. reinhardtii*

Data	Category	Signal	Frequency (%)	Z-score with order-3 Markov model
All sites	Whole	UGUAA	13.39	32.88
		GUGGG	16.10	22.38
		GGUGG	15.16	39.61
		CUGUA	5.95	42.48
		UGCAA	5.00	23.16
Illumina	Whole	UGUAA	13.16	32.31
		GUGGG	17.63	21.02
		GGUGG	16.54	38.02
		GAGGG	11.70	18.02
		CUGUA	5.68	42.64
		UGCAA	4.67	21.36
	5'UTR	UGUAA	13.58	Data too small
	CDS	GGCGG	16.98	3.31
		GCGGG	8.45	3.88
	Intron	GGUGG	47.80	22.33
	3'UTR	UGUAA	24.38	25.87
		CUGUA	9.90	15.32
		UGCAA	7.32	16.93
	Intergenic	GUGGG	19.56	11.04
		GGUGG	18.69	23.50
		GAGGG	12.98	9.66
		GGGAG	12.36	8.82
		UGUAA	8.57	15.10
		GGCGG	10.97	11.03
		GCGGG	10.83	8.82
454	Whole	UGUAA	31.67	21.81
		GUAAC	14.14	10.24
		CUGUA	13.09	10.70
		UGCAA	8.37	16.93
	5'UTR	UGUAA	24.87	Data too small
	CDS	UGUAA	5.50	Data too small
	Intron	UGUAA	34.78	Data too small
	3'UTR	UGUAA	36.88	18.53
		CUGUA	14.96	9.53
		UGCAA	8.93	14.81
	Intergenic	UGUAA	21.12	10.46
ESTs	Whole	UGUAA	45.55	14.60
		GUAAC	19.78	7.43
		CUGUA	17.35	7.39
		UGCAA	8.72	11.12
	5'UTR	UGUAA	23.47	Data too small
	CDS	UGUAA	19.61	Data too small
	Intron	UGUAA	39.73	Data too small
	3'UTR	UGUAA	47.86	13.58
		GUAAC	20.36	6.71
		CUGUA	17.93	6.77
		UGCAA	8.57	10.45
	Intergenic	UGUAA	34.05	4.98

"Data too small" means Z-score cannot be calculated by RSAT because the input sequence number is too small.
