# Supplementary material for: Bioinformatics Analysis of Alternative Polyadenylation in Green Alga Chlamydomonas reinhardtii Using Transcriptome Sequences from Three Different Sequencing Platforms
Source: G3 (Bethesda). 2014 Mar 13;4(5):871–83. doi: 10.1534/g3.114.010249 (PMC4025486; doi:10.1534/g3.114.010249)
Supplement: Supporting Information [file supp_g3.114.010249_TableS2.pdf]

Table S2 The conserved pentamers detected in the FUE regions in *C. reinhardtii*

Data	Signal	Frequency (%)	Z-score with order-3 Markov model
All sites	GUGUG	35.51	None
	GCGGC	28.90	None
	GGUGG	27.35	22.88
	GUGCG	19.93	17.50
	GUGGG	24.43	14.81
	CGUGC	15.73	14.60
	GCGUG	22.72	13.11
	UGUGU	27.19	12.38
	GGCGG	33.30	10.87
Illumina	GUGUG	37.25	None
	GCGGC	29.71	None
	GGUGG	28.75	27.29
	GUGCG	20.19	19.05
	GUGGG	25.59	16.20
	GCGUG	23.16	15.75
	GAGGG	21.27	15.26
454	GUGUG	21.91	None
	GCGUG	21.11	5.23
	GCAUG	19.69	4.14
	GUGCG	18.88	4.46
ESTs	GUGUG	23.87	None
	GCGUG	23.64	3.34
	GGGCC	14.83	3.52
	UGCAU	14.71	4.18

“None” means Z-score was too low to be on the listed generated by RSAT.
