# Supplementary material for: Bioinformatics Analysis of Alternative Polyadenylation in Green Alga Chlamydomonas reinhardtii Using Transcriptome Sequences from Three Different Sequencing Platforms
Source: G3 (Bethesda). 2014 Mar 13;4(5):871–83. doi: 10.1534/g3.114.010249 (PMC4025486; doi:10.1534/g3.114.010249)
Supplement: Supporting Information [file supp_g3.114.010249_FigureS3.pdf]

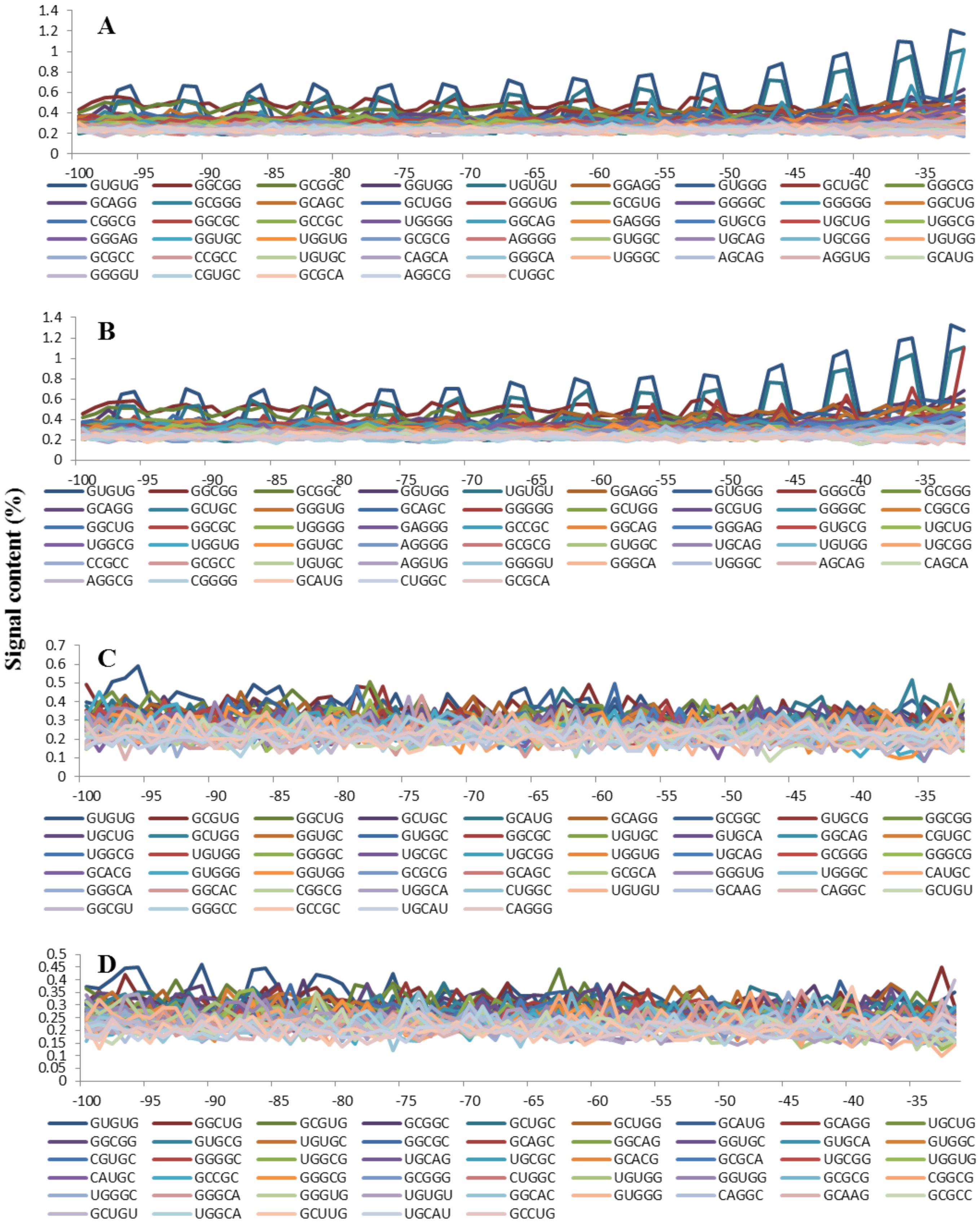

Figure S3 The top frequent motifs from different datasets in the FUE region. (A) All PAC data (including ESTs, 454 and Illumina). (B) Illumina data. (C) EST data. (D) 454 data.
